# Multimodal lifestyle intervention during neoadjuvant chemotherapy in patients with early breast cancer as a strategy to improve treatment response: Protocol of the DIANA randomized clinical trial

**DOI:** 10.1371/journal.pone.0361188

**Published:** 2026-10-27

**Authors:** Helena Castillo, Raquel Sebio-Garcia, Graciela Martinez-Palli, Marina Siso, Miguel Garriz, Esther Sanfeliu, Fara Brasó, Isaac Cebrecos, Inés Torras, Lara Quintas, Raquel Gómez, Sergi Ganau, Berta Díaz-Feijoo, Eduard Mension

**Affiliations:** 1 Department of Gynecology, Hospital Clínic i Provincial de Barcelona (HCPB), Barcelona, Spain; 2 Fundació Clínic per a la Recerca Biomèdica (FCRB) - IDIBAPS, Barcelona, Spain; 3 Department of Physiotherapy and Rehabilitation, Hospital Clínic i Provincial de Barcelona (HCPB), Barcelona, Spain; 4 Department of Anesthesiology, Hospital Clínic i Provincial de Barcelona (HCPB), Barcelona, Spain; 5 Department of Nutrition and Dietetics, Hospital Clínic i Provincial de Barcelona (HCPB), Barcelona, Spain; 6 Department of Psychology, Hospital Clínic i Provincial de Barcelona (HCPB), Barcelona, Spain; 7 Department of Pathology, Hospital Clínic i Provincial de Barcelona (HCPB), Barcelona, Spain; 8 Department of Biology, Hospital Clínic i Provincial de Barcelona (HCPB), Barcelona, Spain; 9 Department of Medical Oncology, Hospital Clínic i Provincial de Barcelona (HCPB), Barcelona, Spain; 10 Department of Radiology, Hospital Clínic i Provincial de Barcelona (HCPB), Barcelona, Spain; Alfaisal University, College of Medicine, SAUDI ARABIA

## Abstract

Breast cancer (BC) represents a major global health concern. Lifestyle factors influence both prevention and prognosis, but evidence on their impact on oncological response to BC treatment remains limited. We present the protocol of a randomized clinical trial designed to assess whether a multimodal intervention—combining physical exercise, psychological support, and nutritional counseling during neoadjuvant chemotherapy (NAC)—could improve pathological response, measured by the Residual Cancer Burden (RCB) index, in women with early BC. A total of 216 women will be randomized (1:1) to control (standard of care, n = 108) or intervention (n = 108). The intervention consists of a supervised, individualized multimodal prehabilitation program that combines physical exercise, psychotherapy, and nutritional support delivered throughout NAC (4–6 months). Tumor pathology, including microenvironment analysis, will be assessed before NAC and after surgery. Data will be collected at baseline, preoperatively, and at 1 and 12 months postoperatively. Long-term oncological outcomes will be reported annually for a minimum of 5 years, with the intention to extend to 10 years. We hypothesize that the intervention group will show higher pathological response rates than controls, with a targeted 20-percentage point improvement in the rate of favorable RCB response (fRCB, defined as RCB class 0 or I), from approximately 40% in the control group to 60% in the intervention group. Additional expected benefits include improved tolerance to NAC, better quality of life, enhanced postoperative recovery, and optimized physical, psychological, and nutritional status. Differences in tumor microenvironment profiles are also anticipated. Ethics approval was granted by the Ethics Committee of the Hospital Clínic de Barcelona (HCB/2023/1036, 26 March 2024). Written informed consent will be obtained from all participants. The DIANA trial will provide evidence on multimodal prehabilitation as a non-invasive, cost-efficient strategy to improve both health and oncological outcomes in BC. Findings will be disseminated via peer-reviewed publications, conference presentations, and patient groups. Trial Registration Number: NCT07107594.

## Introduction

Breast cancer (BC) is the most common neoplasm and the leading cause of cancer death in women worldwide. Neoadjuvant chemotherapy (NAC) is increasingly used in BC treatment due to its capacity to assess tumor response in vivo, which has prognostic value and helps guide adjuvant therapies. Strategies to increase pathological response rates remain insufficiently explored. Emerging evidence indicates that lifestyle factors, including physical exercise, dietary habits, and psychological support, could influence BC prognosis [1].

Physical exercise can reduce mortality in BC patients by 15–48% and may decrease local recurrence [2,3]. Proposed mechanisms include improved tumor oxygenation and enhanced immune response [4]. Physical activity also improves tolerance to chemotherapy, leading to better treatment completion rates [5,6]. Overweight and obesity, reported in 60% of BC patients prior to neoadjuvant regimens [7], are associated with increased rates of local recurrence, overall mortality, cancer-specific mortality [8,9] and worse quality of life in BC [10]. Weight gain is also one of the most frequent adverse effects of treatment (35–85% of BC patients undergoing NAC [11,12]). A healthy balanced diet and maintaining a normal body weight could reduce treatment’s side effects up to 20–30% [13,14], mortality by over 20% and improve quality of life [7,15,16]. Psychological distress affects 20–40% of BC patients, impacting treatment adherence and survival [17]. Providing psychological support during oncological treatment can improve the systemic pro-inflammatory state and the general health of patients [18], reducing side-effects of treatments and recurrence and mortality rates by 40–50% [19–22].

Multimodal prehabilitation, encompassing exercise, nutrition, and psychological interventions, has proven multiple benefits across surgical fields [23–25]. However, evidence in BC remains limited, focusing on physical outcomes and quality of life rather than oncological outcomes [1,26,27].

The DIANA trial hypothesizes that implementing a multimodal prehabilitation program will optimize NAC response by modifying the tumor microenvironment, as well as enhancing overall health. It aims to evaluate whether multimodal prehabilitation combining physical exercise, diet, and psychological support increases pathological response rates in women with early BC undergoing NAC.

## Materials and methods

### Study design

The DIANA trial is a randomized controlled trial with two parallel groups conducted at Hospital Clínic of Barcelona that will include women with non-metastatic BC undergoing NAC. Patients will be randomized 1:1 to the prehabilitation or control group and followed prospectively for 1 year after surgery. Recruitment is scheduled from January 2025 to January 2027. Data will be entered by research staff into the Research Electronic Data Capture (REDCap®) platform via a safe webpage interface. Patients and/or the public were not involved in the design, conduct, reporting, or dissemination plans of this research.

### Participants

Eligible participants include adult women with histologically confirmed non-metastatic BC (cT1-T4, cN0-N3, cM0) who are candidates for NAC. Exclusion criteria include physical contraindications for exercise, pregnancy, other active malignancies, regular physical exercise exceeding 150 minutes per week, prior neoplasm treatment with chemotherapy, and severe psychiatric illness.

### Gender perspective

Male BC accounts for less than 1% of all the cases, with pathophysiology, management, and prognosis that differ substantially from those in women and remain less well characterized. Furthermore, physical exercise programs and dietary needs could differ significantly among genders. Given that the impact of multimodal prehabilitation in BC remains unexplored, it is appropriate to first evaluate its implications in women, who represent most patients with this condition.

### Study parameters and outcomes

The **primary outcome** of the trial is the **pathological tumoral response**, obtained from the histological specimen of the oncological surgery performed after NAC and assessed through the **Residual Cancer Burden (RCB) index**. Unlike pathological complete response (pCR), which is a binary endpoint, the RCB index provides a validated, continuous quantification of residual disease that captures the full spectrum of tumor response to treatment. This allows the detection of clinically meaningful improvements that would be missed by a pCR assessment alone, such as a shift from RCB class III to RCB class II or from RCB class II to RCB class I. The prognostic value of the RCB index has been validated across all BC subtypes and treatment categories, with event-free survival worsening significantly per unit of RCB increase [28]. The four levels of response will be divided into a favorable response profile (fRCB), including RCB 0 and I, and an unfavorable response profile (uRCB), including RCB II and III. Treatment response will also be reported as the rate of pathological complete response (pCR, equivalent to RCB 0). The baseline rate of fRCB in the control arm is estimated at 40%, a conservative assumption based on published RCB distribution data [29–31] that accounts for the inclusion of a significant proportion of luminal tumors, which are associated with lower chemosensitivity. The main objective of this trial will be to improve fRCB rates by 20-percentage points in the intervention group (60% fRCB in the intervention arm vs. 40% in the control arm). This 20-percentage-point improvement in fRCB corresponds to an odds ratio (OR) of 2.25 for favorable response (equivalently, an OR of 0.44 for unfavorable response), and it represents the target effect that the trial is powered to detect. This target is based on emerging evidence suggesting that multimodal lifestyle interventions may improve pathological response during NAC [27,32], combined with the biological plausibility of exercise-induced modulation of the tumor microenvironment.

**Secondary outcomes** will include physical, nutritional, and psychological assessments, oncological parameters, and clinical variables:

- **Physical variables** (assessed before and after NAC, and 1 year after the surgery): anthropometric measurements (weight, height and Body Mass Index), physical activity level (international questionnaire of Physical Activity – IPAQ), hand grip strength test (hand-held dynamometer, measured in kilograms), estimated maximal oxygen uptake (VO2 max) based on the 30-Second Sit-to-Stand Test, change in oxygen consumption (VO2) from baseline to post-neoadjuvant therapy through a Cardiopulmonary Exercise Testing (CPET, the oxygen consumption will be measured in all patients ml/kg/min).
- **Psychosocial variables** (before and after NAC, 1 month and 1 year after the surgery): patient-perceived quality of life (European Organisation for Research and Treatment of Cancer Quality of Life Questionnaire Core 30 validated questionnaire – EORTC QLQ-30), anxiety and depression levels (Hospital Anxiety and Depression Scale validated questionnaire – HADS), mindfulness status (Five Facet Mindfulness Questionnaire – FFMQ), body image perception (Body Image Scale – BIS). To assess patient self-perception following the definitive surgical outcome, the BIS questionnaire will be completed only at 12 months post-surgery.
- **Nutritional variables** (before and after NAC, 1 month and 1 year after the surgery): complete blood analysis (hemogram, nutritional and lipidic profile, Interleukin-6, Insulin-like growth factor 1), adherence to Mediterranean diet (14-Item Mediterranean Diet Adherence Screener – MEDAS14).
- **Tumor microenvironment analysis** (at diagnosis and after NAC): a gene expression analysis will be performed on histological material obtained from the diagnostic biopsy before NAC and from the surgical specimen after NAC. This will include the measurement of the expression of 185 breast cancer-related and immune-related genes including the genes of PAM50 and HER2DX and 7 housekeeping genes using the nCounter platform (Nanostring Technologies, Seattle, USA) and the calculation of the 14-gene immunoglobulin signature (CD27, CD79A, HLA-C, IGJ, IGKC, IGL, IGLV3–25, IL2RG, CXCL8, LAX1, NTN3, PIM2, POU2AF1 and TNFRSF17). The vascular density in the tumor bed will also be reported (immunohistochemical staining for CD31 on tumor or tumor bed sections), and the Tumor infiltrating lymphocytes (TILs) quantified.
- **Clinical variables:** the tolerance to NAC and the postoperative recovery will be evaluated through the following variables: number and severity of the adverse effects of NAC (classification according to the Common Terminology Criteria for Adverse Events, version 5.0), chemotherapy completion rate (ratio of delivered versus planned chemotherapy cycles and dose), hospitalization during NAC (number of hospital admissions prior to surgery, absolute count), post-surgical hospitalization stay (length of hospitalization after oncological surgery), pain control after oncological surgery during the hospital stay (Visual Analogue Scale – VAS), post-operative complications classified based on timing (immediate, early, and late) and severity recorded using the Clavien-Dindo scale, emergency visits, and readmissions.
- **Oncological parameters:** Complete features of the tumor and disease staging will be reported. Oncological treatment will be comprehensively documented, specifying the specific pharmacological agents administered, planned and delivered doses, number of cycles, and any dose reductions or treatment delays, which will be considered as covariates in the final statistical analyses.
- **Other variables:** adherence to each individual component of the prehabilitation intervention and overall satisfaction with the program.

The interventions, outcomes, assessments, and time frame of the trial are summarized in Fig 1. The detailed variables and specific assessment tools are available in S2 Table.

**Fig 1:**
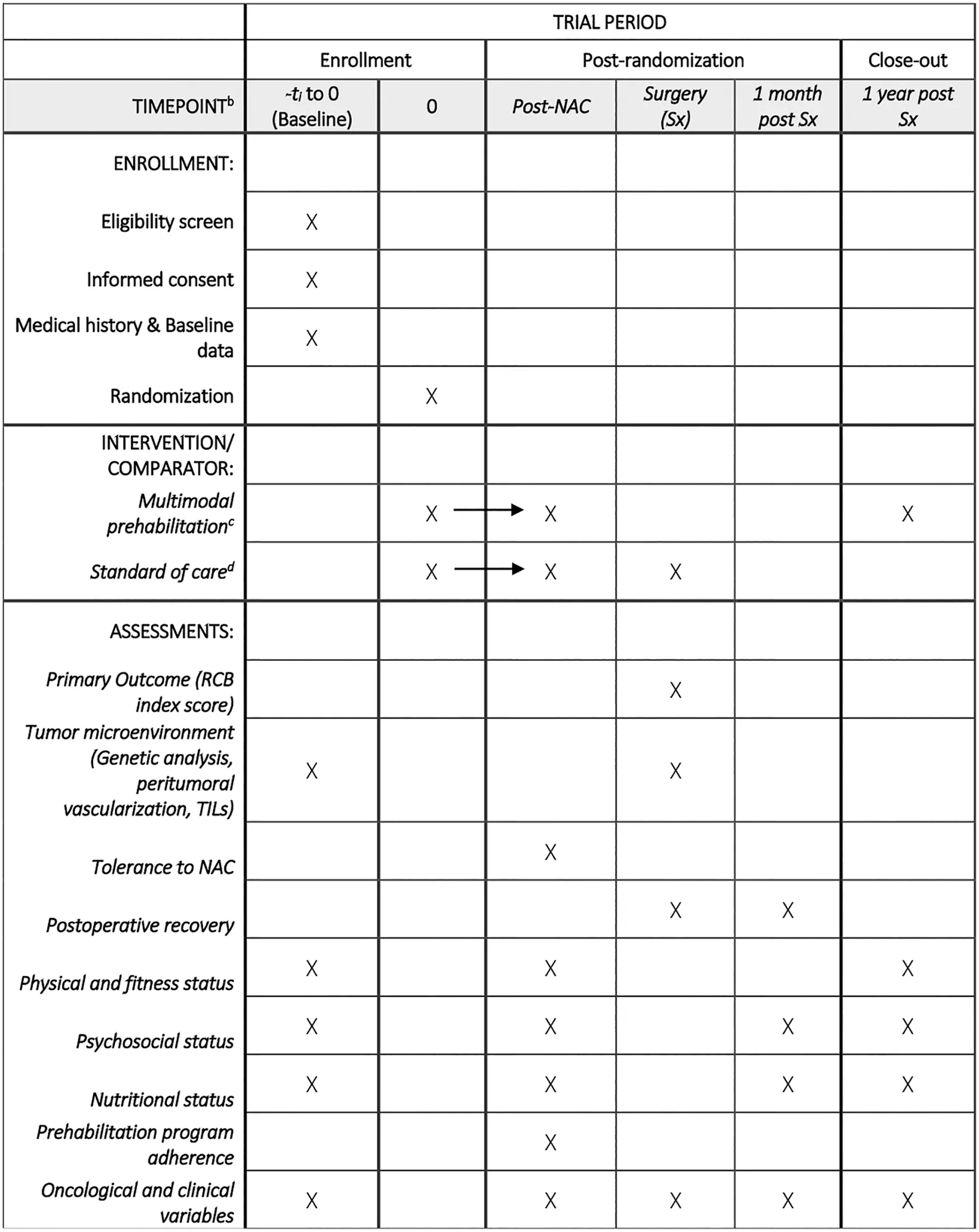
Participant timeline: Schedule of enrollment, interventions, and assessments.

Legend: Abbreviations: NAC: Neoadjuvant Chemotherapy. Sx: Surgery. TILs: Tumor Infiltrating Lymphocytes.

### Sample size calculation, randomization, and blinding

The trial is powered on the dichotomized primary endpoint — favorable RCB response (fRCB, RCB class 0–I) versus unfavorable response (uRCB, RCB class II–III). Assuming a baseline fRCB rate of 40% in the control arm and a target between-group difference of 20 percentage points (fRCB rate of 60% in the intervention arm; OR = 2.25 for favorable response and OR = 0.44 for unfavorable response), a two-sided test of two proportions with an alpha risk of 5% and 80% power requires 97 evaluable patients per arm (194 in total). Allowing for an anticipated 10% loss to follow-up (194/0.9), the target enrollment is 216 patients (108 per arm).

Because RCB is ordinal, the primary endpoint will additionally be analyzed with a proportional-odds model (see Statistical Analyses); using Whitehead’s method [33] with an illustrative control-arm RCB-class distribution consistent with pooled data [29] (22%, 18%, 42% and 18% for RCB 0, I, II and III, respectively), the planned sample retains at least 80% power to detect a common odds ratio of 2.07, reflecting the greater statistical efficiency of the ordinal analysis.

Using a balanced randomization method, patients will be assigned to the intervention (n = 108) or the control group (n = 108) in a 1:1 ratio. Randomization will be stratified according to the tumor immunohistochemical subtype at diagnosis (luminal-like HER2 -, HER2+ and triple negative) and will be performed by the research team using computerized methods (REDCap® software). Assessor-blind masking will be applied by the pathologist and the research team analyzing the results. Unblinding will occur only for medical emergencies or regulatory/safety reasons and will be performed by the principal investigator of the trial.

### Study visits and data collection

Patients will be recruited during their first visit with gynecology following a confirmed diagnosis of non-metastatic BC eligible for NAC. Informed consent will be obtained, and randomization will take place.

All patients from both study groups will undergo a basal Cardiopulmonary Exercise Test (CPET) before starting oncological treatment. Furthermore, they will be offered to register on the Surgifit^®^ digital platform (www.surgifit.es/en), a digital solution specifically designed to support prehabilitation services. It provides access to information on all program components, nutritional guidance, promotion of physical activity, and, only for the prehabilitation group, a content library with videos and documents for performing physical exercises and mindfulness practices at home. It also features a chat for patients to communicate with their professionals, as well as tools to monitor their physical activity in combination with a wearable (smart watch).

Apart from the basal evaluation (prior to the beginning of NAC), study appointments will be scheduled at the end of NAC, 1 month and 1 year after surgery. During these visits, several validated tests and questionnaires regarding their physical, psychosocial, and nutritional status will be completed according to the outcomes of the trial, as outlined in Fig 2. Although the trial concludes at the 1-year postoperative visit, participants will continue to be followed as part of routine clinical care, entering a pre-specified long-term follow-up phase in which disease-free survival, distant recurrence-free survival, and overall survival will be recorded annually for a minimum of 5 years (with the intention to extend to 10 years), ascertained through routine oncological surveillance, electronic health records, and the national mortality registry.

**Fig 2:**
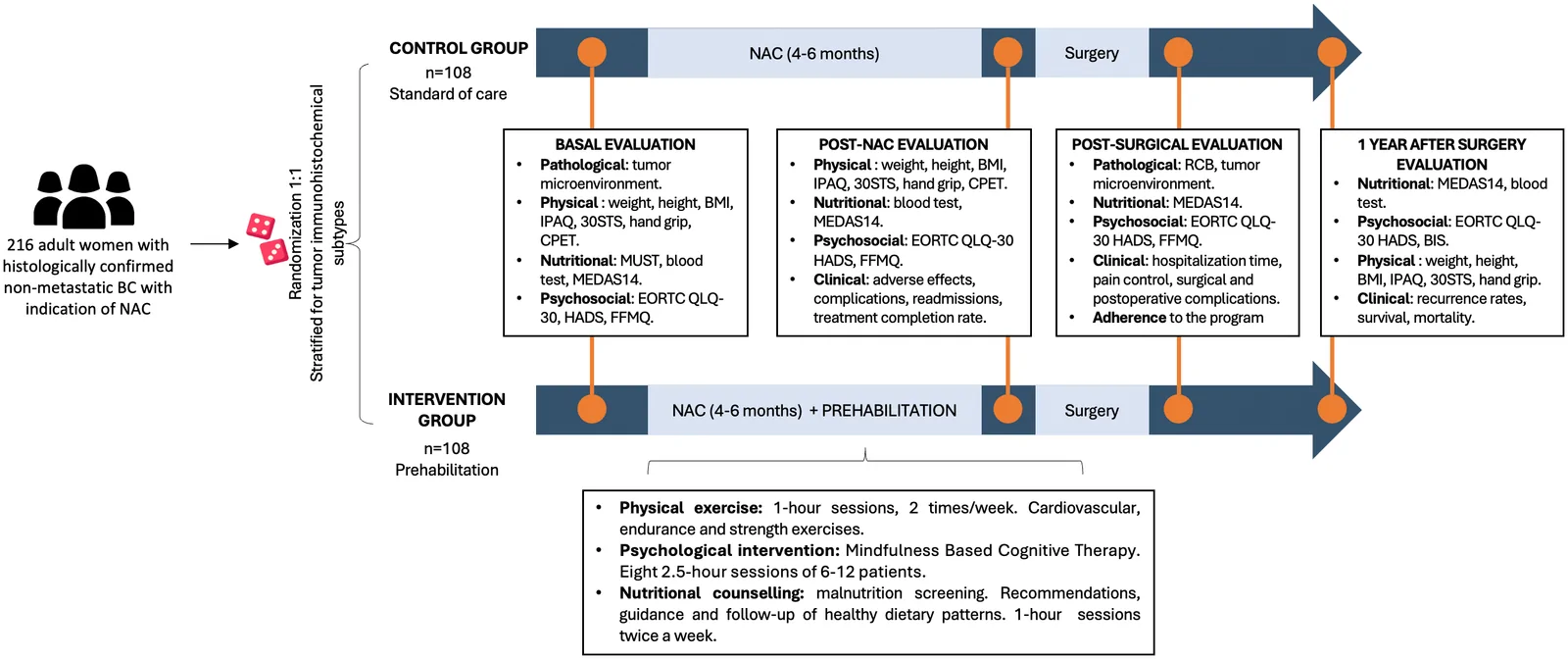
Flow chart of DIANA trial and items assessed in the study visits. Legend: Abbreviations: BC: breast cancer. NAC: neoadjuvant chemotherapy. BMI: body mass index. IPAQ: International Physical Activity Questionnaire. 30STS: 30 Seconds Sit To Stand Test. CPET: Cardiopulmonary Exercise Testing. MUST: Malnutrition Universal Screening Tool. MEDAS-14: Mediterranean Diet Adherence Screener, 14 items. EORTC QLQ-30: European Organization for Research and Treatment of Cancer Quality of Life Questionnaire. HADS: Hospital Anxiety and Depression Scale. FFMQ: Five Facet Mindfulness Questionnaire. RCB: Residual Cancer Burden. BIS: Body Image Scale Questionnaire.

To promote retention, participants will receive regular reminders, flexible scheduling based on each patient’s chemotherapy timetable and personal availability, and telehealth options when feasible. For those discontinuing the trial, outcome data (including primary endpoint and key secondary outcomes) will continue to be collected as per schedule unless consent is withdrawn. All reasons for withdrawal will be documented.

### Intervention: Multimodal prehabilitation

Patients in the intervention group will follow a multimodal intervention program that includes structured physical exercise, psychological intervention, and nutritional counseling during NAC (4–6 months). Participants may discontinue or adjust their allocated intervention at any time for medical or personal reasons, with adaptations documented accordingly.

### Exercise training

Exercise will be delivered in two sequential phases:

1. **Induction phase (first 8–12 weeks)**: supervised one-hour exercise sessions twice weekly in the hospital gym. Each session will include endurance and resistance exercises, followed by a stretching or relaxation segment. Endurance training intensity will be personalized based on baseline Cardiopulmonary Exercise Test (CPET) results, aiming for 25–35 minutes of moderate to high-intensity exercise (40%–85% of VO_2_ peak), with reduced intensity and duration after chemotherapy infusion. Intensity during this phase will be monitored using an ECG monitor (Movesense HR+ sensor, Movesense®, Finland). Resistance training will comprise 5–6 exercises targeting major muscular groups, performed in 2 sets of 10–15 repetitions, with exercise selection adapted to accommodate the presence of a peripheral or central venous access device. Exercise sessions are scheduled individually during the first physiotherapy appointment, which coincides with the baseline CPET, and are not systematically planned on NAC administration days.
2. **Maintenance phase (8–12 weeks):** patients will be asked to continue their exercise program at a local gym located near the hospital and financed by the trial. The structure and periodicity of the workouts will remain as in phase 1. Intensity will be monitored through the modified Borg Scale as well as heart rate and lactate concentration monitors (Onasport, Onalabs®, Spain) and will be increased based on tolerance over the course of NAC. Patients who are unable to access the designated gym for medical or personal reasons will be offered to continue the maintenance phase in the hospital gym.

#### Psychological intervention.

Patients will undergo a complete and structured mindfulness program based on Mindfulness-Based Cognitive Therapy for Cancer (MBCT-Ca), consisting of 8 weekly sessions of 2.5 hours in small groups of 6–12 participants, led by an experienced clinical psychologist within the hospital setting. An alternative intervention consistent of weekly psychoeducational sessions (60–90 minutes) is available for patients who decline mindfulness practice, designed to support emotional adjustment during the intervention period. The schedule for psychological sessions is agreed upon individually during an initial appointment, offered via telehealth when appropriate, and adapted to each patient’s chemotherapy timetable.

#### Nutritional counseling.

Prior to the nutritional intervention, malnutrition screening will be conducted using the validated MUST questionnaire [34]. Patients identified as malnourished will be referred for an individual appointment with a registered dietitian. Patients with adequate baseline nutritional status will receive an initial educational session on healthy eating habits, providing specific dietary recommendations to follow based on the Mediterranean diet pattern [35]. Tailored meal recommendations will be provided to promote a balanced diet, ensuring optimal protein intake according to current guidelines [36] for nutritional therapy in oncology.

### Control group

Standard clinical care during neoadjuvant chemotherapy was chosen as the comparator because it reflects current practice and allows evaluation of the added benefit of multimodal prehabilitation without withholding established treatment. Patients in the control group will continue to receive standard oncological management according to their disease, as well as general advice on healthy dietary habits, information about the benefits of physical exercise and psychological support. This group will also have access to the Surgifit® App, with available general recommendations for a healthy lifestyle and options to register daily physical activity. No restrictions will be imposed on patients’ personal lifestyle choices or additional supportive measures pursued outside the trial. Information on relevant lifestyle behaviors will be systematically collected throughout the trial to account for their potential influence on outcomes.

### Safety considerations

Although no potential harms are expected considering the nature of the suggested intervention, they will be systematically assessed at each study visit. Chemotherapy-related toxicities will be graded with CTCAE v5.0, while exercise-, nutrition-, and psychology-related adverse events will be monitored by the respective specialists and documented in case report forms.

Given the low-risk nature of the intervention, no independent data monitoring committee has been established. Safety monitoring will be conducted by the principal investigator and study steering committee. All serious adverse events will be reported to the Ethics Committee and Sponsor in accordance with regulations. In addition, the trial will not be independently monitored given the features of the intervention suggested; oversight will be maintained by the steering committee.

### Statistical analyses

Normality will be assessed with the Shapiro-Wilk test, and descriptive statistics will summarize patient characteristics. The primary efficacy analysis will assess the association of the multimodal prehabilitation intervention with fRCB by comparing the two trial groups using a multivariable logistic regression model including treatment allocation, breast cancer immunohistochemical subtype, and tumor stage as covariates, at a two-sided significance level of 0.05. This approach is intended to improve precision and account for baseline differences in expected pathological response rates across biologically distinct breast cancer populations with different baseline tumor burden.

As RCB is an ordinal outcome, the full four-level RCB-class distribution (0, I, II, III) will additionally be compared between arms using a proportional-odds ordinal logistic regression model, adjusted for immunohistochemical subtype and tumor stage, yielding a common odds ratio for the shift across the ordered response categories. The proportional-odds assumption will be assessed (score/Brant test); if it is not satisfied, a partial proportional-odds model will be reported alongside the pre-specified analysis at the fRCB/uRCB cut-point. The RCB class distribution and other categorical secondary outcomes will be compared between groups using the chi-squared test or Fisher’s exact test as appropriate. Continuous outcomes, including physical, nutritional, and psychosocial variables, will be compared using Student’s t-test or the Wilcoxon test depending on the normality of their distribution.

All secondary analyses will be considered exploratory. Adherence to each individual component of the intervention will be quantified throughout the trial, and pre-specified exploratory dose–response analyses will relate cumulative intervention exposure to pathological response. Pre-specified exploratory subgroup analyses according to immunohistochemical subtype will be conducted to evaluate potential heterogeneity of treatment effect across subtypes. Additional exploratory sensitivity analyses according to neoadjuvant treatment category (chemotherapy alone, immunotherapy-containing, and anti-HER2-containing regimens) will be performed to assess the robustness of the findings across different therapeutic backbones. Analyses will follow an intention-to-treat approach, including all randomized patients in their assigned groups.

A planned interim analysis will be performed after half of the target enrollment is reached to evaluate preliminary results and assess for early identification of any emerging trends or concerns, ensuring the study proceeds in alignment with ethical and scientific standards.

Long-term outcomes recorded beyond the trial period during routine oncological surveillance will be analyzed using Kaplan–Meier estimates, log-rank tests, and Cox models adjusted for immunohistochemical subtype and stage, and interpreted as exploratory.

Considering the non-invasive nature of the intervention, no formal statistical stopping rules for efficacy or futility have been pre-specified. Nevertheless, the trial may be terminated early if the interim analysis reveals unforeseen safety concerns or if feasibility is significantly compromised. Missing data will be handled using multiple imputation under the missing-at-random assumption. Sensitivity analyses will assess the robustness of results to different assumptions. The extent of missing data will be reported for each outcome.

All statistical analyses will be conducted using STATA software, version 19.0 (StataCorp LLC, USA).

### Study timeline

The DIANA trial is currently in the recruitment phase, having commenced enrollment in January 2025. Patient recruitment is estimated to be completed by 2027. An interim analysis will be conducted once approximately 50% of the planned sample size (n = 108) has been reached. Final study results are projected to be published in 2028. Long-term survival endpoints will continue to be collected annually for a minimum of 5 years thereafter, with the intention to extend follow-up to 10 years.

### Ethics and dissemination

The protocol has been approved by the Ethics Committee of the Hospital Clinic de Barcelona (HCB/2023/1036, 2024-03-26) and will be conducted in accordance with the Declaration of Helsinki and Good Clinical Practice guidelines. Written informed consent will be obtained from all participants by trained clinicians (oncologists, gynecologists, or research nurses) prior to enrollment, using approved consent forms. Participant confidentiality will be safeguarded by assigning coded identifiers and storing all data in secure, password-protected databases accessible only to the research team. Participants will continue to receive standard oncological care throughout and after the trial. No additional post-trial care is planned beyond routine clinical follow-up.

The full trial protocol will be available as supplementary material with the published manuscript and on ClinicalTrials.gov (NCT07107594, 2025-08-06, www.clinicaltrials.gov/study/NCT07107594?term=diana%20trial&rank=3). De-identified participant data (including data dictionary) and statistical code will be available from the corresponding author upon reasonable request, for academic research purposes, and subject to approval by the study steering committee in accordance with General Data Protection Regulation requirements. Any important protocol modifications (e.g., eligibility criteria, outcomes, analyses) will be communicated to investigators, trial registry (ClinicalTrials.gov), ethics committee, and journals as required.

The results of the DIANA trial will be disseminated through presentations at national and international scientific meetings, publications in peer-reviewed open-access journals, and engagement with relevant patient associations. A lay summary will also be provided to participants and stakeholders. No identifiable data will be released.

## Discussion

The DIANA trial aims to evaluate the impact of a multimodal prehabilitation program based on non-pharmacological lifestyle interventions on oncological treatment response and overall health status in patients with BC.

Multimodal prehabilitation has been extensively studied in surgical patients with several cancers including colorectal [24], esophagogastric [23] and lung cancer [25], among others. In BC, although some studies on the advantages of unimodal prehabilitation have been published, there are currently no randomized trials evaluating the role of multimodal prehabilitation in BC patients or its potential impact on the response to oncological treatments [26,37,38].

Given that exercise, psychological support and a dietary optimization have individually demonstrated improvements in prognosis and oncological outcomes in BC patients, their combination may produce synergistic benefits, particularly in the context of NAC. Beyond the anticipated oncological benefits, the intervention may enhance patient’s overall health status and reduce the risk of lifestyle-related comorbidities, improve tolerance to oncological treatment, reduce treatment-related toxicities, and promote sustained healthy lifestyle behaviors. Multimodal prehabilitation is further expected to enhance postoperative recovery and reduce the incidence of surgical and chemotherapy-related complications.

At the molecular level, the interplay between lifestyle modifications and the tumor microenvironment represents an emerging area of research. Tumor-infiltrating lymphocytes (TILs), key predictors of response to NAC, are linked to BC prognosis, being an independent predictor of tumoral response after NAC [39–41]. Exercise has been shown to modulate immune responses by altering cytokine levels and lymphocyte subtypes, potentially improving cancer outcomes [42–44]. It may also enhance tumor perfusion and reduce hypoxia through systemic effects such as increased cardiac output, arterial pressure, and vascular adaptation, which could enhance the efficacy of cancer treatments [4,45]; in preclinical models of BC, these changes were accompanied by an improved response to chemotherapy at an unchanged cytotoxic dose [45].

Several ongoing clinical trials are currently evaluating the role of prehabilitation in BC. Nevertheless, most of them suggest a unimodal approach based exclusively on physical activity (NCT05184582, NCT05216302, NCT06309290, NCT06360809, NCT04623554, NCT05716542, NCT05976815, KCT0008961) or a bimodal strategy omitting psychological intervention (NCT02786875, NCT06266312, NCT03131024), and others focus on the feasibility of the program (NCT05901142, ACTRN12622000584730) rather than oncological outcomes.

The DIANA trial therefore represents the first randomized clinical trial designed to evaluate the impact of multimodal prehabilitation on pathological response to neoadjuvant systemic treatment in BC, alongside clinical outcomes, quality of life and tumor microenvironment. A key strength of our study is that training will be entirely personalized, with both training volume and intensity meticulously recorded during the study. Furthermore, all patients will participate in a structured mindfulness training program.

The present study will also have some limitations. The primary endpoint is assessed within the trial period, which concludes at the 1-year postoperative visit (16–18 months from enrollment, comprising 4–6 months of chemotherapy and 1 year after surgery). Within this timeframe, recurrences and long-term survival cannot yet be evaluated, which is particularly relevant given the prolonged recurrence risk in both ER-negative and ER-positive disease. Furthermore, pathological response is a validated but surrogate endpoint; although RCB is strongly prognostic across subtypes, whether an intervention-induced improvement in RCB translates into a long-term survival benefit has not been established for this class of intervention. For these reasons, and although it falls outside the trial period, the protocol now incorporates a pre-specified commitment to extended long-term follow-up (disease-free, distant recurrence-free, and overall survival for a minimum of 5 years, with the intention to reach 10 years), so that the clinical data generated by DIANA can be fully interpreted regardless of the primary result.

Another limitation that warrants emphasis is the fact that the trial is powered to detect a moderate-to-large effect on pathological response. Should the true effect be smaller, this single-center study may be underpowered, and a smaller but still clinically relevant benefit would require confirmation in a larger, ideally multicenter, trial.

It has also been considered the possible Hawthorne effect in the control group: participating in a trial about the potential benefits of a lifestyle intervention could lead to higher levels of physical activity, healthier eating patterns or the implementation of anxiety-control techniques in these patients. Additionally, variability in NAC regimens across patients represents a potential source of confounding, as response rates may differ according to the specific agents, doses, and number of cycles administered. However, stratified randomization by immunohistochemical subtype — which largely determines the standard regimen — is expected to ensure balanced regimen distribution across groups. Multivariable adjustment and subgroup sensitivity analyses will further mitigate this potential bias. Moreover, it should be noted that the effectiveness of the suggested intervention is strongly linked to adherence to the program, and high adherence rates could be difficult to achieve in a population generally physically inactive and undergoing a chemotherapy treatment. To minimize these potential bias or confounding factors, patients in both groups will wear smartwatches enabling us to ascertain the level of physical activity performed throughout the study period. Additionally, both the multimodal intervention and digital support are intended to enhance program adherence. Finally, the unicentric nature of the trial is a limitation itself.

## Conclusions

The benefits of lifestyle interventions, combining physical exercise, psychotherapy, and nutritional support have been demonstrated across various pathologies; however, evidence in BC remains limited despite its status as one of the most prevalent malignancies worldwide. The DIANA trial aims to provide robust evidence on multimodal prehabilitation as a non-invasive, cost-efficient strategy to optimize oncological outcomes, quality of life and physical and mental health in BC patients.

## Supporting information

S1 File SPIRIT 2025 checklist of items to address in a randomized trial protocol. (DOCX)

S2 Table Summary of primary and secondary outcomes, assessment tools, and evaluation timeline of the DIANA trial. Abbreviations: NAC: Neoadjuvant Chemotherapy; RCB: Residual Cancer Burden; FFPE: Formalin-Fixed Paraffin-Embedded; IGG: Immunoglobulin; TILs: Tumor-Infiltrating Lymphocytes; CTCAE: Common Terminology Criteria for Adverse Events; Sx: Surgery; VAS: Visual Analogue Scale; BMI: Body Mass Index; IPAQ: International Physical Activity Questionnaire; CPET: Cardiopulmonary Exercise Testing; VO2: Oxygen Consumption; EORTC QLQ-30: European Organization for Research and Treatment of Cancer Quality of Life Questionnaire; HADS: Hospital Anxiety and Depression Scale; FFMQ: Five Facet Mindfulness Questionnaire; BIS: Body Image Scale; MEDAS-14: Mediterranean Diet Adherence Screener.
(PDF)

S3 File Copy of the protocol that was approved by the ethics committee and official certificate of approval (English translation). (PDF)
