# Supplementary material for: Multimodal lifestyle intervention during neoadjuvant chemotherapy in patients with early breast cancer as a strategy to improve treatment response: Protocol of the DIANA randomized clinical trial
Source: PLoS One. 2026 Oct 27;21(10):e0361188. doi: 10.1371/journal.pone.0361188 (PMC13712585; doi:10.1371/journal.pone.0361188)
Supplement: Supplementary file 1 [file pone.0361188.s002.pdf]

	Outcome	Variables	Description	Time frame					
				Basal	NAC	Post-NAC	Surgery (Sx)	1m-post Sx	1y-post Sx
Primary	Tumor pathological response	Residual Cancer Burden Index (RCB)	Assessed from the histological specimen of the oncological surgery performed after NAC.				X		
Secondary	Tumor microenvironment	Gene expression analyses	Gene expression analysis of 185 breast cancer and immune-related genes (including PAM50, HER2DX, and a 14-gene IGG signature) using the nCounter platform on Formalin-Fixed Paraffin-Embedded (FFPE) tumor tissue.	X			X		
		Vascular density in peritumoral areas	Immunohistochemical staining for CD31 on tumor or tumor bed sections.	X			X		
		Tumor infiltrating lymphocytes (TILs)	Quantification of TILs in the histological tumor samples (diagnostic biopsy and surgical specimen).	X			X		
	Tolerance to NAC	Adverse effects of NAC	Classification according to the CTCAE (Common Terminology Criteria for Adverse Events) version 5.0.			X			
		Chemotherapy completion rate	Ratio of delivered versus planned chemotherapy cycles and dose.			X			
		Hospitalization during NACT	Number of hospital admissions prior to surgery (absolute count).			X			
	Postoperative recovery	Post-surgical hospitalization	Length of hospitalization after oncological surgery (days).				X	X	
		Pain control after oncological surgery	Assessment through the Visual Analogue Scale (VAS) during the hospital stay.				X		
		Post-operative complications	Classified based on timing (immediate, early, and late). Severity recorded using the Clavien-Dindo scale. Emergency visits and readmissions will also be reported.				X	X	
	Physical and fitness status	Anthropometric variables	Weight (kg), height (m), and Body Mass Index (kg/m2)	X		X			X
		Physical activity level	Evaluated using the international questionnaire of Physical Activity (IPAQ).	X		X			X
		Strength	Assessed through the hand grip strength test, using a hand-held dynamometer (measured in kilograms).	X		X			X
		Estimated maximal oxygen uptake (VO2 max)	The 30-Second Sit-to-Stand Test will be performed and recorded as an absolute number.	X		X			X
		Change in oxygen consumption (VO2) from baseline to post-neoadjuvant therapy.	Through a Cardiopulmonary Exercise Testing (CPET), the oxygen consumption will be measured in all patients (ml/kg/min).	X		X			
	Psychosocial status	Patient-perceived quality of life	Measured using the EORTC QLQ-30 validated questionnaire.	X		X		X	X
		Anxiety and Depression levels	Assessed using the Hospital Anxiety and Depression Scale (HADS).	X		X		X	X
		Mindfulness status	Assessed through the Five Facet Mindfulness Questionnaire (FFMQ).	X		X		X	
		Body image perception	Evaluation through the validated questionnaire Body Image Scale (BIS).						X
	Nutritional status	Blood test with nutritional profile	Complete blood analysis: Including liver and renal profile, blood count, ionogram, albumin, prealbumin, glucose, interleukin-6 and IGF-1.	X		X			X
		Adherence to Mediterranean diet	Validated Questionnaire of Mediterranean Diet Adherence (MEDAS-14).	X		X		X	X
	Prehabilitation program adherence		Number of sessions attended relative to the scheduled sessions.			X			
	Oncological and clinical variables	Several variables regarding the tumor type, staging and characteristics, comorbidities of the patient, detailed neoadjuvant treatment, surgical specifications, adjuvant therapies, recurrence, survival, mortality, etc.		X	X	X	X	X	X

Table 1. Summary of primary and secondary outcomes, assessment tools, and evaluation timeline of the DIANA trial.

Abbreviations: NAC: Neoadjuvant Chemotherapy; RCB: Residual Cancer Burden; FFPE: Formalin-Fixed Paraffin-Embedded; IGG: Immunoglobulin; TILs: Tumor-Infiltrating Lymphocytes; CTCAE: Common Terminology Criteria for Adverse Events; Sx: Surgery; VAS: Visual Analogue Scale; BMI: Body Mass Index; IPAQ: International Physical Activity Questionnaire; CPET: Cardiopulmonary Exercise Testing; VO2: Oxygen Consumption; EORTC QLQ-30: European Organization for Research and Treatment of Cancer Quality of Life Questionnaire; HADS: Hospital Anxiety and Depression Scale; FFMQ: Five Facet Mindfulness Questionnaire; BIS: Body Image Scale; MEDAS-14: Mediterranean Diet Adherence Screener.
