# Supplementary material for: Multimodal lifestyle intervention during neoadjuvant chemotherapy in patients with early breast cancer as a strategy to improve treatment response: Protocol of the DIANA randomized clinical trial
Source: PLoS One. 2026 Oct 27;21(10):e0361188. doi: 10.1371/journal.pone.0361188 (PMC13712585; doi:10.1371/journal.pone.0361188)
Supplement: Supplementary file 2 [file pone.0361188.s003.pdf]

1 General Information

1.1 Study Identification

Title: Multimodal intervention during neoadjuvant chemotherapy in patients with non-metastatic breast cancer as a strategy to improve pathological response to treatment: DIANA trial (multimodal prehabilitation: DIet, ANxiety control psychotherapy, physical Activity).
Version and date: DIANA_v3. 02/25/2024

1.2 Sponsor identification

Eduard Mension Coll. Graduate in Medicine and Surgery, Attending Physician in Gynecology and Obstetrics. Doctor of Medicine and Surgery. Gynecology / ICGON / Hospital Clínic de Barcelona. mension@clinic.cat

1.3 Identification of principal investigators at our center:

Eduard Mension Coll. Graduate in Medicine and Surgery, Attending Physician in Gynecology and Obstetrics. Doctor of Medicine and Surgery. Gynecology / ICGON / Hospital Clínic de Barcelona. mension@clinic.cat

Helena Castillo Espín. Graduate in Medicine and Surgery, Attending Physician in Gynecology and Obstetrics. Master of Science. Gynecology / ICGON / Hospital Clínic de Barcelona. hcastillo@clinic.cat

Berta Díaz Feijoo. Graduate in Medicine and Surgery, Attending Physician in Gynecology and Obstetrics. Doctor of Medicine and Surgery. Gynecology / ICGON / Hospital Clínic de Barcelona. bdiazfe@clinic.cat

Graciela Martínez Pallí. Graduate in Medicine and Surgery, Attending Physician in Anesthesiology and Resuscitation. Doctor of Medicine and Surgery. Anesthesiology / Surgical Department / Hospital Clínic de Barcelona. gmartin@clinic.cat

1.4 Identification of collaborating researchers from participating centers

Montserrat Muñoz Mateu. Graduate in Medicine and Surgery, Senior Consultant in Medical Oncology. Doctor of Medicine and Surgery. Medical Oncology / ICMHO / Hospital Clínic de Barcelona. mmunoz@clinic.cat

Blanca González Farre. Graduate in Medicine and Surgery, Senior Specialist in Pathological Anatomy. Doctor of Medicine and Surgery. Pathological Anatomy / Biomedical Diagnostic Center (CDB) / Hospital Clínic de Barcelona. mbgonzal@clinic.cat

Raquel Sebio García. Graduate and Doctor of Physical Therapy, Associate Professor. Rehabilitation / Institute of Medical and Surgical Specialties / Hospital Clínic de Barcelona. sebio@clinic.cat

Betina Campero Calzada. Graduate in Human Nutrition and Dietetics, Dietitian-Nutritionist. Endocrinology and Nutrition /ICMDM / Hospital Clínic de Barcelona. bcampero@clinic.cat

Marta García Moliner. Graduate in Psychology and Nursing, Nurse and Psychotherapist, General Health Psychologist. Gynecology / ICGON / Hospital Clínic de Barcelona. garcia18@clinic.cat

*Hospital Clínic de Barcelona: C. Villarroel, 170, 08036 Barcelona. Telephone: 932 27 54 00.

2 Justification

Breast cancer (BC) is the most common cancer worldwide and the leading cause of death in women. Neoadjuvant chemotherapy (NAC) is currently one of its main therapeutic pillars, given its ability to assess the pathological response of the tumor in vivo, which has prognostic value, in addition to determining subsequent adjuvant treatments. The pathological response after NACT can be assessed using the residual cancer burden (RCB) index, which has been shown to be a good predictor of prognosis in BC, both in terms of survival and recurrence. (Lancet Oncol. 2022 Jan;23(1):149-160; BMC Cancer. 2024 Jan 2;24(1):13. Ann Surg Oncol. 2022 Dec;29(13):8060-8069).

In recent years, lines of research have emerged focusing on the impact of environmental factors and lifestyle changes on CM, such as physical exercise, diet, and psychological support, especially in the surgical setting. These actions are part of what is known as prehabilitation, defined as the set of strategies applicable prior to surgery to optimize the physical and emotional health of patients. Improving preoperative physical condition through these programs has been shown to be effective in reducing postoperative complications and speeding up patient recovery.

In parallel with the generation of evidence in the surgical field, it has recently been demonstrated that the application of each of these strategies individually in patients with MC improves oncological outcomes and even has a positive effect on quality of life and tolerance to medical treatments (Chen Y et al. Future Oncology. 2018;14(15):1487-1496. Andersen BL et al. Cancer. 2008;113(12):3450-3458. Oh PJ et al. Psychol Health. 2016;31(4):396-419. Ochoa-Arnedo C et al. Int J Clin Health Psychol. 2022;22(2):100303. Peterson LL et al. Curr Oncol Rep. 2018;20(7):50. Cannioto RA et al. J Natl Cancer Inst. 2021;113(1):54-63. Van Waart H et al. J Clin Oncol. 2015;33(17):1918-1927). In addition, evidence is available suggesting the potential of such interventions to favorably modulate the tumor microenvironment through a decrease in intratumoral hypoxia, as well as an increase in the antitumor immune response and a modulation of the systemic proinflammatory state (Wiggins JM et al. Exerc Sport Sci Rev. 2018;46(1):56-64. Valenza C et al. Cancers. 2023;15(3):767. Denkert C et al. Lancet Oncol. 2018;19(1):40-50).

Despite the proven benefits of prehabilitation in various fields, comprehensive care for cancer patients is underrepresented and there is little evidence regarding its impact on the response to systemic treatments in patients with MC. There is a lack of solid evidence assessing the role of multimodal prehabilitation (combination of different prehabilitation strategies) in CM, nor has its impact on response and tolerance to cancer treatment been evaluated (Toohey et al. Breast Cancer Res Treat. 2023;197(1):1-37).

The current study proposes the application of a multimodal prehabilitation program during NAC window based on structured physical exercise, psychological support, and nutritional advice in patients with CM as a strategy to increase treatment response through changes in the tumor microenvironment. On the other hand, it will be evaluated whether the intervention provides better treatment tolerance and better postoperative recovery for patients.

3 Study hypothesis

The application of a multimodal prehabilitation program based on structured physical exercise, psychological support, and nutritional counseling during neoadjuvant chemotherapy will optimize oncological outcomes in patients with breast cancer, resulting in an increase in the tumor pathological response rate. Likewise, this intervention will have the ability to modulate the tumor microenvironment, modifying the tumor-infiltrating lymphocytes (TILs) quantitatively and/or qualitatively and the peritumoral vascularization. The multimodal intervention will provide additional benefits for patients, such as improved tolerance to oncological treatment, improved quality of life, and optimized postoperative recovery and physical condition.

4 Objective and Purpose of the

Study Main objective

To evaluate whether a multimodal prehabilitation program based on physical exercise, diet, and psychological support during neoadjuvant chemotherapy in women with non-metastatic breast cancer produces an increase in pathological tumor response after cancer treatment in this group of patients.

Secondary objectives

As secondary objectives, the impact of the multimodal prehabilitation program will be assessed on:

- The tumor microenvironment: gene expression analysis to evaluate genes related to immunity and breast cancer. Quantitative assessment of peritumoral vascularization before and after intervention.

- Tolerance to systemic treatment with NAC: recording of complications and adverse effects, NAC completion rate, and admission rate during treatment.

- Postoperative recovery: collection of mean hospital stay, surgical and postoperative complications, pain control, and rate and reason for readmissions in both groups.

- The psychosocial sphere of patients. Quality of life parameters, psychological status, and anxiety levels will be assessed using validated questionnaires.

- The physical condition of the patients: changes in maximum and submaximal cardiorespiratory capacity, as well as changes in daily physical activity and bioimpedance.

- Nutritional parameters: changes in the patients' nutritional profile assessed analytically, as well as changes in eating patterns and dietary habits.

In addition, patients' experiences of participating in the study and their level of adherence to the program will be assessed.

4.1 Primary and secondary variables

Primary variable: The primary variable of the trial is the pathological tumor response obtained in the histological specimen from breast and/or axillary surgery performed after neoadjuvant chemotherapy. This response will be assessed using the RCB (*Residual Cancer Burden*) index, which evaluates six histological parameters that refer to the presence or absence of residual disease in the breast and/or axilla, as well as its quantification, after chemotherapy treatment. The four response levels of the RCB index will be divided into a favorable response profile (fRCB), which will include RCB categories 0 and I, and an unfavorable response profile (uRCB), which will include RCB II and III.

Secondary variables:

- *Anatomopathological variables*: assessment of the tumor microenvironment and peritumoral vascularization in all patients using the following approach:

  - **Assessment of the tumor microenvironment:** gene expression analysis will be performed at the end of systemic treatment. This will include measurement of the expression of 185 genes related to breast cancer and the immune system, including PAM50 and HER2DX genes, as well as 7 reference genes, using the nCounter platform (Nanostring Technologies, Seattle, USA) and calculation of the immunoglobulin signature of 14 genes ([D27, CD79A, HLA-C, IGJ, IGKC, IGL, IGLV3-25, IL2RG, CXCL8, LAX1, NTN3, PIM2, POU2AF1, and TNFRSF17]).

  - **Assessment of vascularization density** in both the peritumoral area of diagnostic biopsies and the tumor bed in surgical specimens. The assessment will be performed by evaluating microvascular density using immunohistochemical staining for CD31 in tumor or tumor bed sections, through a two-step evaluation. First, *hotspot* areas will be selected using a 4x magnification optical microscope, and then all vascular lumens present in that field will be counted manually using 20x magnification.

- *Clinical variables*:

  - **Chemotherapy completion rate**. The proportion of patients who have received all chemotherapy cycles at the corresponding dose will be recorded. This will be assessed prior to surgery in both groups.

  - **Need for hospitalization during neoadjuvant chemotherapy**. This will be recorded before surgery as the absolute number of hospitalizations.

  - **Adverse effects of neoadjuvant chemotherapy**. These will be recorded by the research team and classified according to the CTCAE (Common Terminology Criteria for Adverse Events) version 5.0 terminology. They will be collected at the end of NAC in both groups.

  - **Length of hospital stay** after cancer surgery (counted in days).

  - **Pain control** after cancer surgery (assessment according to the Visual Analog Scale – VAS).

  - **Complications**: these will be classified according to their timing as immediate postoperative

immediate (during the procedure or 24 hours after), intermediate (up to 7 days after surgery), and late postoperative (7 to 30 days after surgery). Their severity will be recorded according to the Clavien-Dindo scale.

- **Emergency room visits and readmissions**: absolute number, reason, and days of hospital admission. These will be recorded up to 30 days after surgery.

- *Physical variables*: these will be recorded at baseline, at the end of neoadjuvant chemotherapy treatment (between 1-2 weeks later), and 1 year after surgery.

  - **Height, weight, and body mass index**.

  - **Maximal and submaximal cardiorespiratory capacity**. These will be assessed using incremental cardiopulmonary exercise testing (CPET). The different parameters derived from the test will be collected, as well as the concentration of lactate in sweat during exercise, determined non-invasively (Onasport™, Onalabs®). CPET will only be performed before and after neoadjuvant therapy, without being repeated one year after surgery.

  - **Heart rate variability (HRV).** Patients will be instructed to monitor their HRV using a photoplethysmography device (Garmin® model vivosmart™ or vivoactive™). To this end, they will be asked to lie on their back for 3 to 5 seconds in the morning upon waking up. Any variations in HRV related to neoadjuvant treatment will be recorded, as well as the response to the exercise sessions performed in the intervention group.

  - **Level of physical activity**. This will be recorded using the validated IPAQ (International Physical Activity Questionnaire) and the average number of steps per day collected using the same photoplethysmography device mentioned in the previous variable.

  - **Muscle** *strength* will be measured using the hand *grip test*.

- *Psychosocial variables*: these will be recorded at baseline, at the end of neoadjuvant chemotherapy treatment, one month after surgery, and one year after surgery.

  - **Quality of life** as perceived by patients using the EORTC QLQ-30 questionnaire.

  - **Anxiety and depression** in patients using the *Hospital Anxiety and Depression Scale (HADS).*

  - Mindfulness capacity: using the *Five Facet Mindfulness Questionnaire (FFMQ).*

  - Self-perception of body image: using the validated *Body Image Scale (BIS)* questionnaire, which will be assessed 1 year after surgery.

- *Nutritional variables*: these will be recorded at baseline, at the end of neoadjuvant chemotherapy treatment, one month after surgery, and one year after surgery.

- **Blood test with nutritional profile**: this will include a determination of the patients' liver and kidney profile, as well as a complete blood count, ionogram, albumin, prealbumin, ammonium, insulin, glucose, IGF-1, and IL-6.

- **Adherence to the Mediterranean diet**: using the validated MEDAS-14 questionnaire.

- *Other variables*:

  - Record of **adherence** to the prehabilitation program (i.e., number of training sessions attended by the patient in relation to the number of scheduled sessions, proper completion of nutritional supplementation, mindfulness practices/week).

5 Study design

This is a randomized, controlled, single-center, national, masked (single-blind for the team evaluating the samples at the pathological level) clinical trial with two parallel groups (intervention group and control group, respectively).

Randomization will be performed in a balanced manner with a 1:1 ratio using STATA software, version 15.1 (StataCorp LLC, USA).

6 Selection of participants

Patients eligible for inclusion in this study are women with a confirmed diagnosis of non-metastatic breast cancer and an indication for neoadjuvant chemotherapy prior to surgery at the Breast Pathology Unit of Hospital Clínic de Barcelona.

Eligible patients will be recruited during their first gynecological hospital visit at our center following a confirmed diagnosis of non-metastatic breast cancer eligible for neoadjuvant chemotherapy treatment.

6.1 Subject inclusion criteria

Patients must meet **all** of the following **inclusion criteria**:

- Confirmed histological diagnosis of non-metastatic breast cancer: stages cT1-T4 cN0-N3 M0 candidates for neoadjuvant chemotherapy treatment.

- Age ≥ 18 years.

- Signed informed consent.

6.2 Subject exclusion criteria

Patients who meet **any** of the following **exclusion criteria** will be excluded from the trial:

- Contraindication or physical inability to perform moderate/high-intensity physical exercise.
- Pregnant or breastfeeding patients.
- Other active neoplasms occurring synchronously.
- Metastatic breast cancer (stage IV).
- Patients who already engage in regular physical exercise: ≥150 minutes of moderate or intense aerobic exercise per week.

7 Treatment and study schedule

Patients will be recruited at their first gynecology visit after a confirmed diagnosis of non-metastatic breast cancer eligible for neoadjuvant chemotherapy (visit 1). At this visit, patients will be given the informed consent form and will be randomized to either the intervention group or the control group. All patients will undergo a baseline physical assessment (anthropometric variables, level of physical activity using the IPAQ questionnaire, cardiorespiratory stress test, *hand grip* strength test), nutritional assessment (blood test with nutritional profile, MEDAS-14 questionnaire), and psychosocial assessment (EORTC QLQ-30, FFMQ, and HADS questionnaires). In addition, the tumor microenvironment (gene expression and vascularization test) will be studied in the diagnostic biopsy sample of all patients.

Patients randomized to **the intervention group** will follow a multimodal prehabilitation program based on physical exercise, psychological support, and nutritional counseling during NAC (approximately 4 to 6 months).

- <u>Physical exercise</u>: Initial phase: Two weekly sessions of one hour each of structured physical exercise supervised by physical therapists in a hospital setting during the first eight weeks. Maintenance phase: Subsequently, and until the 4-6 months of NAC treatment are completed, patients will be referred to StudioNova to continue physical training. StudioNova and our hospital, through the team of researchers involved in this proposal, have a collaboration agreement. One of the researchers (RS), a physical therapist specializing in physical training for cancer patients, will act as a liaison between the two centers and will supervise both the complexity and intensity of the exercises, which will be progressively adapted to the different phases of the study and treatment. To this end, heart rate and lactate production will be monitored non-invasively during training (Onasport™, Onalabs®).

- <u>Psychological support</u>: a comprehensive and structured Mindfulness-Based Cognitive Therapy for Cancer (MBCT-Ca) program, consisting of eight weekly 2.5-hour sessions in groups of 6 to 12 participants, led by a clinical psychologist with experience in the hospital setting. An alternative intervention will be available for patients who reject the practice of mindfulness, consisting of weekly psychoeducational sessions (60 to 90 minutes), designed to support emotional adjustment during the prehabilitation period.

- Nutritional counseling: Before the nutritional intervention, malnutrition will be screened using the validated MUST questionnaire. If malnutrition is detected, the specific circuit will be activated. Patients with adequate nutritional status at baseline will receive an initial information session on healthy eating habits, providing specific dietary recommendations based on the Mediterranean diet pattern. Personalized meal recommendations will be offered to promote a balanced diet, ensuring optimal protein intake according to current guidelines for nutritional therapy in oncology.

During the first training session, patients will be registered on the Surgifit®* digital platform, which will be specially adapted for monitoring the program. The platform provides access to information related to all components of the program. Patients will have access to nutritional advice and healthy recipes, as well as information on physical activities to perform and a library of videos and support documents for doing the exercises at home. The platform also allows for mindfulness practices. It also has a chat system that will allow patients to contact the relevant professionals at the Clinic, as well as monitor and track their physical activity. It also allows for the remote completion of the various follow-up questionnaires.

**Note: The Surgifit® digital environment consists of a backend for professionals and a mobile app for patients. To register the patient anonymously on the platform, a generic email address (estudiodiana@gmail.com) will be created, and each patient will be assigned an alias based on their study identifier. For example, if the identifier is DIANA0001, that patient's email address in the app will be estudiodiana+DIANA001@gmail.com. The patient is registered in the app with the anonymous ID and not with their real name. The platform is located on Amazon Web Services cloud servers, with the server located in Frankfurt, Germany. Health Circuit's role is that of "Platform Provider," responsible for security rules, implementations, and platform availability. AWS's role is that of cloud service provider for hosting the platform. Data stored on the servers will be encrypted using AES-256, and encryption keys will be stored separately. The servers will use firewalls and other techniques to block unauthorized calls and brute force attacks, while monitoring systems will be in place to detect unusual behavior. Data will be stored for at least 5 years, renewable to 10 years.*

The purpose of the mobile application is to provide feedback to the patient on their progress throughout the prehabilitation program. Specifically, patients will have access to: i) scheduled visits related to the prehabilitation program; ii) monitoring of their physical activity level (via a monitoring device provided by the hospital); iii) monitoring of body weight and compliance with prescribed nutrition guidelines; and iv) mindfulness exercises for managing stress related to the cancer process and planned surgery. Patients will also receive nutritional and physical activity advice and may be asked to complete follow-up questionnaires related to physical activity, nutrition, or mood.

Patients in **the control group** will follow the usual clinical management without undergoing the multimodal prehabilitation program. In accordance with current recommendations, they will be given general advice on healthy dietary habits and informed of the benefits of physical exercise, without specific follow-up or monitoring. They will attend the relevant appointments in accordance with the usual schedule for their condition.

In addition to the usual visits for their pathology, which can be coordinated with these, the patients included in the study will be assessed at the end of neoadjuvant chemotherapy treatment and one month after surgery. During these visits, the relevant tests and questionnaires described in the following figure will be carried out.

Time	Procedure
BASELINE STATUS (BEFORE NAC)	· Eligibility check<br>· Signing of informed consent<br><br>· **Pathological assessment**: analysis of tumor microenvironment in diagnostic biopsy sample.<br>· **Physical assessment**: anthropometric variables (weight, height, BMI), IPAQ questionnaire, cardiopulmonary exercise test, hand grip.<br>· **Nutritional assessment**: blood test with nutritional profile, MEDAS-14.<br>· **Psychosocial assessment**: EORTC QLQ-30 questionnaire, FFMQ, and HADS.
RANDOMIZATION	**Intervention**: multimodal prehabilitation during NAC (4-6 months of physical exercise + psychotherapy + nutritional counseling).
	**Control**: usual care
NAC	At the end of treatment:<br>· **Physical assessment**: anthropometric variables (weight, height, BMI), IPAQ questionnaire, cardiopulmonary exercise test, hand grip.<br>· **Psychosocial assessment**: EORTC QLQ-30 questionnaire, FFMQ, and HADS.<br>· **Clinical assessment**: recording of adverse effects, complications, readmissions, treatment completion rate.
SURGERY AND ADMISSION	· **Anatomopathological assessment**: histological analysis of the surgical specimen and quantification of tumor pathological response (RCB). Analysis of tumor microenvironment in surgical specimen.<br>· **Clinical assessment**: average length of hospital stay, pain control (VAS), surgical complications.<br>· **Nutritional assessment**: blood test with nutritional profile, MEDAS-14.
POSTOPERATIVE	1 month post-surgery:<br>· **Psychosocial assessment**: EORTC QLQ-30, FFMQ, and HADS questionnaires.<br>· **Nutritional assessment**: MEDAS-14.<br>· **Clinical assessment**: record of postoperative complications.<br>· Assessment of adherence to the prehabilitation program.<br><br>1 year post-surgery:<br>· **Physical assessment**: anthropometric variables (weight, height, BMI), IPAQ questionnaire, hand grip.<br>· **Nutritional assessment**: blood test with nutritional profile, MEDAS-14.<br>· **Psychosocial assessment**: EORTC QLQ-30 questionnaire, HADS, BIS.<br>· Patient Reported Experience Measures: patients' perceived experience of the trial.

Figure. Flowchart of study visits and their content.

8 Statistics

8.1 Sample size

It is estimated that the average rate of a favorable RCB index (fRCB), which includes RCB 0 and RCB I, will be approximately 40% in the control group. Therefore, unfavorable RCB rates (uRCB), which include RCB II and RCB III, are expected to be 60% (Yau C et al. The Lancet Oncology. 2022;23(1):149-160; Xu X et al. BMC Cancer. 2024;24(1):13. Wang W et al. Gland Surg. 2021;10(12):3211-3221; Elder EA, et al. Ann Surg Oncol. 2022;29(13):8060-8069). The primary objective of this trial will be to improve response rates by 20% in the intervention group, reaching 60% fCRB and 40% uCRB rates.

With 80% power and a 5% alpha risk, a sample size of 194 patients is estimated, increasing to 214 patients taking into account an expected loss of 10%. Using a balanced randomization method, patients will be assigned to the intervention group (n=107) or the control group (n=107) in a 1:1 ratio. Randomization will be stratified according to the histological subtype of the tumor at diagnosis (luminal-like HER2-, HER2+, and triple negative) and will be performed by the research team using computerized methods. Blinding will be applied by the pathologist and the research team that will analyze the results.

8.2 Statistical analysis

Statistical analyses will be performed using STATA software, version 15.1 (StataCorp LLC, USA). The normal distribution of the sample will be assessed using the Shapiro-Wilk test. Continuous variables will be compared using the T-test for independent or paired samples and will be presented as mean ± standard deviation. Contingency tables will be evaluated using Fisher's exact test. A $p<0.05$ will be considered statistically significant.

9 Ethics and legal aspects

This study will be conducted in compliance with the Declaration of Helsinki (current version; currently Fortaleza, Brazil, October 2013). The study will be conducted in accordance with the protocol and the relevant legal requirements under Law 14/2007 of July 3 on biomedical research.

Informed consent will be requested from patients before their inclusion in the study.

10 Data management

Each patient's data will be coded and collected in an encrypted and anonymized Redcap® database and will be processed solely and exclusively within the framework of their participation in this study. In accordance with data protection legislation, the women recruited may exercise their rights of access, modification, opposition, and cancellation of data, for which they must contact the principal investigator of the study. Access to your personal information will be restricted to the principal investigator of the study and their collaborators, the Clinical Research Ethics Committee, and authorized personnel, when necessary to verify the study data and procedures, but always maintaining the confidentiality of such data in accordance with current legislation. Only data collected for the study will be transmitted to third parties, and in no case will this data contain information that could directly identify you, such as your name and surname, initials, address, social security number, etc. If such a transfer occurs, it will be for the same purposes as those described in the study and confidentiality will be guaranteed.

11 Data Processing and Record Archiving. Data Confidentiality.

The processing, communication, and transfer of the personal data of all participants will comply with Regulation (EU) 2016/679 of the European Parliament and of the Council of April 27, 2016, on the protection of natural persons with regard to the processing of personal data and on the free movement of such data, and Organic Law 3/2018 of December 5th on the Protection of Personal Data and Guarantee of Digital Rights. The legal basis for the processing of your data is the consent you give in this document, in accordance with the provisions of Article 9 of EU Regulation 2016/679.

The data collected for these studies will be identified only by a code, so no information that could identify participants will be included. Only the study physician and his or her collaborators with access to the source data (medical records) will be able to link the data collected in the study to the patient's medical records.

The identity of the participants will not be available to any other person except in the event of a medical emergency or legal requirement.

Health authorities, the Research Ethics Committee, and personnel authorized by the study sponsor may have access to personally identifiable information when necessary to verify study data and procedures, but always maintaining confidentiality in accordance with current legislation.

Only encrypted data will be transferred to third parties and other countries, and under no circumstances will this data contain information that could directly identify the participant (such as name and surname, initials, address, social security number, etc.). If such a transfer takes place, it will be for the same purpose as the study described and confidentiality will be guaranteed.

If encrypted data is transferred outside the EU, whether to entities related to the hospital where the patient is participating, to service providers, or to researchers collaborating with us, the participants' data will be protected by safeguards such as contracts or other mechanisms established by the data protection authorities.

As project promoters, we undertake to process data in accordance with EU Regulation 2016/679 and, therefore, to keep a record of the processing activities we carry out and to perform a risk assessment of the processing we carry out, in order to know what measures, we will have to apply and how to do so.

In addition to the rights already covered by previous legislation (access, modification, opposition, and cancellation of data, deletion in the new Regulation), participants can now also limit the processing of data collected for the project that is incorrect, request a copy, or request that it be transferred to a third party (portability). To exercise these rights, they should contact the principal investigator of the study or the Data Protection Officer at Hospital Clínic de Barcelona at protecciodades@clinic.cat.Así. They also have the right to contact the Data Protection Agency if they are not satisfied.

The data cannot be deleted even if a patient leaves the study, in order to guarantee the validity of the research and comply with legal obligations and drug authorization requirements.

The Investigator and the Sponsor are required to retain the data collected for the study for at

least 10 years after its completion. Subsequently, personal information will only be retained by the center for healthcare purposes and by the sponsor for other scientific research purposes if the patient has given their consent and if permitted by applicable law and ethical requirements.

12 Management of biological samples

In the current study, three biological samples will be collected per patient:

- Diagnostic biopsy: breast lesion biopsy performed for the diagnosis of breast cancer. This sample is taken from all patients due to their underlying pathology, not for the specific purposes of this trial. However, an additional tumor microenvironment analysis will be performed on all biopsy samples from participating patients (see previous sections). Biopsy samples will be analyzed in the Pathology Department. They will then be stored following the usual healthcare protocol.

- Nutritional profile analysis: this will be performed on patients at baseline, after completing chemotherapy, and after surgery in order to assess the nutritional profile of women during the study period and after the interventions performed. They will be analyzed at the Central Laboratory of Hospital Clínic de Barcelona.

- Surgical specimen: resection specimen obtained after breast cancer surgery. As with the surgical biopsy, this sample is taken from all patients due to their underlying pathology, not for the specific purposes of this trial. An additional analysis of the tumor microenvironment will be performed on all surgical specimens from participating patients (see previous sections). The analysis will be performed in the Pathology Department. Subsequently, they will be stored following the usual healthcare protocol.

13 Funding

The following grants have been awarded to fund this research:

- *EMILI LETANG – JOSEP FONT* CLINICAL RESEARCH CONTRACTS AND *CLINICAL – LA* P*EDRERA* RESEARCH CONTRACT

- TV3 MARATHON GRANT 2024 – SEXUAL AND REPRODUCTIVE HEALTH

14 Publication Policy

The study sponsor, together with the research team, is committed to publishing the results of the study, whether positive or negative.

15 Specific risks of the study

Participation in the does not entail any additional risks beyond those of standard clinical practice to patients, as they will receive the usual standard of care. All patients will follow the current care protocols in terms of treatment or follow-up after confirmation of the definitive diagnosis. The intervention proposed in this study does not involve any invasive procedures or pharmacological or surgical treatments other than those relevant to the patients' underlying condition, consisting exclusively of lifestyle modifications and psychological support.
