# Supplementary material for: Multimodal lifestyle intervention during neoadjuvant chemotherapy in patients with early breast cancer as a strategy to improve treatment response: Protocol of the DIANA randomized clinical trial
Source: PLoS One. 2026 Oct 27;21(10):e0361188. doi: 10.1371/journal.pone.0361188 (PMC13712585; doi:10.1371/journal.pone.0361188)
Supplement: Supplementary file 4 [file pone.0361188.s005.docx]

**RESPONSE TO REVIEWERS**

The authors would like to thank the Editor and the reviewers for their constructive feedback and technical checks. This letter provides a point-by-point response to all comments, along with the corresponding modifications made to the revised manuscript.

**Journal requirements**

In response to the journal requirements, we have carefully revised the manuscript to ensure compliance with PLOS ONE style and formatting guidelines:

- The Funding Statement has been amended to declare all sources of financial support and now includes the sentence "There was no additional external funding received for this study." Grant numbers have been verified and reconciled across the Funding Information and Financial Disclosure sections. Funding-related text has been removed from the manuscript.
- The ethics statement has been confirmed to appear exclusively within the Ethics and Dissemination subsection, which forms part of the Materials and Methods section of the manuscript.
- A copy of Table 1 (S2 Table) has been included in the revised manuscript as requested.

**Response to the Editor’s comments:**

“Several sections use conversational wording and occasional redundancy. Consider replacing with more formal alternatives and consolidating repeated claims”.

- We thank the Editor for this observation. We have carefully reviewed the manuscript and revised sections containing conversational wording or redundant phrasing, replacing them with more formal and concise alternatives. For instance, we replaced "Nowadays, strategies to increase response rates remain unexplored" with "Strategies to increase pathological response rates remain insufficiently explored"; replaced informal phrasing such as "it is convenient to first study" with "it is appropriate to first evaluate" in the Gender perspective section; and consolidated repeated mentions of the anticipated intervention benefits across the Discussion into a single, streamlined paragraph. All specific changes are highlighted in the revised manuscript with tracked changes.

“Your statistical plan includes a log-rank test (time-to-event), but the protocol’s primary and listed outcomes are mainly cross-sectional/ordinal/categorical (RCB class, pCR, questionnaire scores, biomarkers, etc.). Please explain/clarify”.

- We thank the Editor for identifying this inconsistency. As the Editor correctly notes, the primary and secondary outcomes of the DIANA trial are predominantly categorical, ordinal, or continuous variables, for which the log-rank test is not appropriate. The sentence has been removed from the revised manuscript and replaced with a clarification of the correct statistical approaches for each type of outcome.

“Some inconsistency was noted. You state an “estimated 20% improvement in RCB index scores,” but later you operationalize the primary endpoint as fRCB rate improving from ~40% to 60%. Thank you”

- We agree with the editor that the phrasing "estimated 20% improvement in RCB index scores" in the Abstract is imprecise and potentially ambiguous. This has been corrected in the revised manuscript to ensure consistency with the operationalization of the primary endpoint throughout the protocol. The Abstract now reads "an estimated 20-percentage point improvement in fRCB rates, from approximately 40% in the control group to 60% in the intervention group", which is consistent with the sample size calculation and the primary endpoint definition described in the Methods section.

**Response to the Reviewer’s comments:**

Reviewer #1

“The main outcome of the trial is supposed to be the pathological tumoral response. Although the interventions described would most likely affect tumoral response, the problem of many different types of drugs that can be used for NAC (neoadjuvant chemotherapy), responses to which can be varied depending on the type of breast cancer being treated and the individual patient. Also, there could be variations in doses, number of treatments, etc. This could be a strong confounding factor. How are the proposers planning to account for this?”

- We thank Reviewer #1 for raising this important point. We fully acknowledge that variability in NAC regimens represents a potential confounding factor, and several strategies have been incorporated into the trial design to address this. First, only patients receiving cytotoxic neoadjuvant chemotherapy will be eligible; excluding regimens based on endocrine therapy or CDK4/6 inhibitors. Second, all administered regimens will be comprehensively documented, including pharmacological agents, doses, and administration schedules. Regarding tumor subtype variability, randomization is stratified by immunohistochemical subtype (luminal-like HER2−, HER2+, and triple-negative), which ensures balanced distribution of the main subtypes — and, by extension, their associated standard regimens — across both arms. As a result, the proportion of patients receiving subtype-specific agents (e.g., pembrolizumab in triple-negative breast cancer or trastuzumab/pertuzumab in HER2-positive tumors) is expected to be comparable between groups. For the primary endpoint analysis, a multivariable logistic regression model will be performed incorporating treatment allocation, immunohistochemical subtype, and tumor stage as covariates. Since immunohistochemical subtype inherently captures both regimen-related information and intrinsic chemosensitivity differences, adjusting by subtype is more methodologically appropriate than adjusting by NAC regimen directly. The robustness of findings across different therapeutic backbones will be further assessed through pre-specified exploratory sensitivity analyses stratified by NAC treatment category (chemotherapy alone, immunotherapy-containing, and anti-HER2-containing regimens). These considerations have been clarified in the revised manuscript.

“It is stated that randomization will be stratified according to the tumor histological subtype, which would be another confounding factor. What exactly does this "stratification" mean? The secondary outcomes may be less affected by the type of NAC except for physical variables, since different NAC would have different physical side effects”.

- Stratified randomization by immunohistochemical subtype means that, at the time of randomization, patients are allocated to the intervention or control group separately within each tumoral subtype stratum (luminal-like HER2−, HER2+, and triple-negative breast cancer). This ensures that each subtype is represented in comparable proportions across both arms, regardless of the overall enrollment sequence. This stratification is particularly relevant in the context of this trial, since HER2-positive and triple-negative disease, although less prevalent in the general breast cancer population, are disproportionately represented among candidates for NAC and are associated with distinct chemotherapy regimens and response rates. By balancing tumor subtype distribution between groups, stratified randomization controls for both the biological heterogeneity of the tumor and the regimen-related variability inherent to each subtype, thereby reducing the risk of confounding in the primary endpoint analysis. Regarding the secondary outcomes, we agree with the reviewer that physical variables may be particularly susceptible to regimen-specific toxicity profiles. For this reason, all administered NAC regimen details will be systematically documented and incorporated in exploratory sensitivity analyses stratified by treatment category (chemotherapy alone, immunotherapy-containing, and anti-HER2-containing regimens), as described in the revised statistical plan.

“It is not clear when the supervised one-hour exercise sessions in the hospital gym will take place; are these sessions being done on the NAC administration day? If so, the day will be longer than usual for patients, and perhaps more difficult after administration of the NAC. However, asking patients to come to the hospital on days not scheduled for NAC might be difficult, thus limiting accrual to the trial. The same consideration exists for the psychological intervention sessions - are they held on a different day, same day as NAC? Both may prove to be difficult for patients”.

- We thank Reviewer #1 for raising this fundamental concern, which was carefully considered and discussed during the study design phase.

Rather than imposing a fixed prehabilitation schedule, the trial has been deliberately designed around a flexible, patient-centered approach to maximize adherence and comfort for patients, since the ultimate aim of the trial is to improve the treatment for breast cancer patients and their overall health.

For the exercise sessions, scheduling is discussed individually with each patient during the first physiotherapy appointment, which coincides with the baseline CPET assessment performed prior to the start of NAC, thereby combining clinical assessments and program planning in a single visit. Exercise sessions are therefore not systematically scheduled on NAC administration days, as this would be physically demanding and logistically burdensome for patients.

The same flexible approach applies to the psychological and nutritional interventions, for which an initial appointment — offered via telehealth when appropriate — is held with the corresponding specialist to agree on the most suitable schedule for each patient individually. This coordination is facilitated by continuous communication across the multidisciplinary team, including oncologists, specialized nurses, physiotherapists, psychologists, and dietitians. This clarification has been incorporated into the revised manuscript.

“In the maintenance phase, patients will be asked to continue their exercise program at a local gym. Will gym membership be paid by the trial? What if patients don't have access to a local gym?”

- We appreciate Reviewer #1 for this practical observation. We would like to clarify that gym membership costs during the maintenance phase are fully covered by the trial, so no financial burden is placed on participants. Furthermore, the local gym is located in close proximity to the hospital (approximately 450 meters, a 5-minute walk), which was deliberately chosen to minimize logistical barriers for patients and promote retention. The facility is staffed by physical trainers with specific expertise in oncological patients, ensuring that the continuity and quality of the supervised training is maintained throughout the maintenance phase. Patients who are unable to access the designated gym for medical or personal reasons will be offered to continue the maintenance phase in the hospital gym. This clarification has been incorporated into the revised manuscript.

Reviewer 2

“The authors present a multimodal complex intervention trial to improve outcomes in women receiving NAC for breast cancer. The suggestion is that this intervention will improve pathological response rates as measured by the residual burden score. The motivation is clear but the suggested improvements are perhaps somewhat overstated especially as these are women who are more likely to die from breast cancer, and the EBCTCG results on 200,000 women published at SBCS suggest a 20% reduction is distant recurrence is going to be associated with a difference in BMI of at least 12 points. The effect on breast cancer mortality is what is important here and this may also be driven by better adherence but given that it is reliably determined that the effect on breast cancer mortality is about 2/3 of that on distant recurrence the effects discussed here are of the order of those of adjuvant chemotherapy.”

- We thank Reviewer #2 for this thoughtful comment. The authors would like to clarify a fundamental distinction regarding the endpoints being discussed. The EBCTCG analysis presented at SABCS 2024 (Pan et al., Abstract GS2-09) examines the observational association between BMI at diagnosis and long-term outcomes in breast cancer patients, such as distant recurrence and breast cancer mortality. Importantly, this analysis does not evaluate the effect of BMI reduction as an intervention on these outcomes – it describes a prognostic association, not a causal interventional effect. While we fully acknowledge the considerable statistical power afforded by the large sample size and the scientific rigor of the EBCTCG group, the findings should nonetheless be interpreted with appropriate caution, given both the observational nature of the data and the fact that the work has, to date, been disseminated as a conference abstract rather than a full peer-reviewed publication. The primary endpoint of the DIANA trial is pathological tumor response at the time of surgery, as measured by the validated RCB index, a surrogate endpoint that is biologically and temporally distinct from distant recurrence or mortality. The DIANA trial is therefore not claiming a mortality benefit equivalent to that of adjuvant chemotherapy but rather hypothesizing an improvement in a short-term surrogate oncological endpoint that is potentially modifiable by biological mechanisms acting within the timeframe of NAC.

“The justification for stopping follow-up so soon needs to be given especially as the risk of recurrence in ER -ve disease persists for 5 years, and that for ER positive disease for 20 years. Patient groups have repeatedly stressed the importance of long-term follow-up and we do not know if response rates are a good surrogate for this particular intervention.”

- The authors completely agree with Reviewer #2 that long-term outcomes such as disease recurrence and mortality are of paramount clinical importance in breast cancer research, and we acknowledge that recurrence risk persists for many years, particularly in ER-positive disease. However, assessing these outcomes as primary endpoints falls outside the scope of the current trial. The primary aim of the DIANA trial is to evaluate the short-term impact of a multimodal lifestyle intervention on pathological tumor response – hypothetically mediated through modulation of the tumor microenvironment – as measured by the RCB index in the surgical specimen obtained after NAC completion. The 1-year postoperative follow-up was deliberately included to assess whether the multimodal prehabilitation program may promote sustained lifestyle changes beyond the intervention period, and to capture medium-term physical, nutritional, psychological, and quality-of-life outcomes. While recurrence and survival data will be collected at this timepoint, they are not the primary focus of the current trial. Regarding the use of RCB as a surrogate endpoint, it is worth noting that RCB is a well-validated prognostic tool with demonstrated association with long-term survival outcomes across breast cancer subtypes. We fully acknowledge, however, that whether RCB improvement translates into long-term survival benefit in the context of a lifestyle intervention specifically requires confirmation, and extended long-term follow-up of DIANA trial participants is planned as a future research priority. This has been clarified in the revised manuscript.

“The effect size here to change from 60% RCB2-3 to 40% RCB2-3 needs to be justified; the associated odds ratio is 0.67 which is quite large for a short term intervention. This effect size is larger than the well known KEYNOTE trials which initially appears implausible.”

- We thank Reviewer #2 for this methodologically important observation. We acknowledge that the proposed effect size might appear to be ambitious; however, we would like to provide a comprehensive justification supporting its plausibility from multiple angles.

First, regarding the primary endpoint itself, it is important to highlight that the RCB index represents a considerably richer and more sensitive outcome measure than pCR alone. Unlike pCR, which is a binary endpoint capturing only the presence or absence of residual disease, the RCB index quantifies the full continuum of pathological tumoral response by incorporating the two-dimensional area of residual invasive cancer, the cellularity of the tumor bed, and the nodal burden. This means that clinically meaningful improvements in tumor response – such as a shift from RCB class III to RCB class II, or from RCB class II to RCB class I – are captured and contribute to the primary endpoint, even when they do not result in a complete pathological response. The prognostic relevance of this continuum is well established: data from the I-SPY2 randomized clinical trial (JAMA Oncol. 2021 Nov 1;7(11):1654-1663, n=938) demonstrated that RCB was consistently and independently prognostic across all breast cancer subtypes and treatment categories, with event-free survival worsening significantly per unit of RCB increase in every subtype. Critically, that study also showed that effective investigational treatments shifted the entire distribution of RCB toward lower values, not only increasing pCR rates, and that this shift was associated with improved event-free survival in an exploratory analysis. The use of fRCB (RCB 0-I) as the primary endpoint in DIANA therefore captures a broader and more sensitive spectrum of treatment benefit than pCR alone, which supports the plausibility of detecting a significant effect with the proposed sample size.

Second, the baseline assumption of 40% fRCB in the control arm is deliberately conservative. Based on the pooled analysis by Yau et al. (Lancet Oncol. 2022 Jan;23(1):149-160; n=5,161), the global RCB distribution after NAC is approximately 45% RCB 0-I and 55% RCB II-III across all subtypes. Given that the DIANA population will include a significant proportion of luminal tumors, which are associated with lower chemosensitivity and pCR rates, a baseline estimate of 40% fRCB is justified and may even be an underestimate of the true proportion of unfavorable responses

Third, the most directly comparable clinical evidence supports the plausibility of the hypothesized effect size. The LEANer trial (J Clin Oncol. 2023 Dec 1;41(34):5285-5295) demonstrated a 25-percentage point increase in pCR rates (53% vs. 28%, p=0.037) in 72 breast cancer patients that underwent NAC assigned to a combined exercise and nutritional intervention, a larger effect than the 20-percentage point improvement in fRCB hypothesized in DIANA. A systematic review and meta-analysis of 30 studies on prehabilitation during neoadjuvant therapy (Support Care Cancer. 2024 Oct 28;32(11):749) further concluded that prehabilitation may improve pathological response and treatment completion compared to standard care. Additionally, the Neo-ACT trial (NCT05184582, n=712) was specifically designed to detect a 10% improvement in pCR with a prehabilitation strategy based on exercise alone during NAC, corroborating the biological plausibility of exercise-driven improvements in pathological response even with a unimodal approach. The DIANA intervention is multimodal, combining physical exercise, nutritional counseling, and psychological support, and may therefore produce synergistic effects exceeding those of any single component.

Fourth, regarding the comparison with the KEYNOTE trials, while definitely informative, this analogy is not directly applicable. Those trials evaluate the addition of pembrolizumab, a potent pharmacological immunotherapy agent acting through a specific immune checkpoint mechanism, whereas DIANA evaluates a non-pharmacological lifestyle intervention acting through multiple distinct and complementary biological pathways, including exercise-induced tumor vascular normalization and improved drug delivery, modulation of antitumor immune responses through enhanced NK and CD8+ T cell activity, and reduction of systemic inflammation and oncogenic hormonal signaling. These mechanisms are biologically plausible and supported by both preclinical and emerging clinical evidence, and their combined effect in the context of a truly multimodal intervention has not yet been evaluated in a rigorous randomized trial.

The corresponding clarifications have been made in the revised manuscript.

“The issue of receptor status is important here as this is highly prognostic and the potentially predictive nature of the marker has not been discussed in the issue of response rates - higher numbers of ER+ women with high Ki-67 may lead to lower response rates. What NAC regimens are allowed and how is the use of drugs like pembrolizumab allowed for in the randomization/eligibility?”

- We thank Reviewer #2 for raising this important point. We fully agree that receptor status is a fundamental aspect to take into consideration in our trial, since it is a key prognostic and predictive factor, and that the proportion of ER-positive tumors with high Ki-67 in the study population may influence overall response rates, given their well-known lower chemosensitivity. Regarding permitted NAC regimens, only patients receiving cytotoxic neoadjuvant chemotherapy will be eligible; regimens based exclusively on endocrine therapy or CDK4/6 inhibitors are excluded from the trial. Since the addition of targeted or immunotherapy agents is determined by tumor biology and constitutes an indispensable component of current standard of care in BC treatment, the use of pembrolizumab in triple-negative breast cancer and trastuzumab/pertuzumab in HER2-positive disease is permitted. All administered treatments will be comprehensively documented, including pharmacological agents, doses, and administration schedules. Randomization is stratified by immunohistochemical subtype (luminal-like HER2−, HER2+, and triple-negative), ensuring balanced distribution of the main subtypes — and, by extension, their associated standard regimens — across both arms. For the primary endpoint analysis, a multivariable logistic regression model will be performed incorporating treatment allocation, immunohistochemical subtype, and tumor stage as covariates. Since immunohistochemical subtype inherently captures both regimen-related information and intrinsic chemosensitivity differences, adjusting by subtype is more methodologically appropriate than adjusting by NAC regimen directly. The robustness of findings across different therapeutic backbones will be further assessed through pre-specified exploratory sensitivity analyses stratified by NAC treatment category (chemotherapy alone, immunotherapy-containing, and anti-HER2-containing regimens). These considerations have been clarified in the revised manuscript.

“The intervention is complex, and it is unclear why the design does not allow the different components to be analysed separately or indeed whether any one component affects the acceptability/adherence for the others.”

- We appreciate the methodological observation from Reviewer #2. The decision not to adopt a factorial design was deliberate and based on several well-founded considerations.

First, the primary scientific hypothesis of the DIANA trial is that the synergistic combination of physical exercise, nutritional counseling, and psychological support produces an effect on pathological tumor response that exceeds what any individual component could achieve in isolation. This is not an arbitrary choice: multimodal prehabilitation combining these three components has already demonstrated benefit across various surgical oncology fields [31, 32, 33], and is currently considered the recommended prehabilitation approach in these settings. Evidence in breast cancer, while more limited, also supports the multimodal strategy [1, 23], although no randomized trial has yet evaluated it specifically during NAC, which is precisely the gap that DIANA aims to address. Testing the multimodal intervention as a whole is therefore not a design limitation, but the deliberate and scientifically justified primary question of this trial.

Second, a factorial design would require at least four study arms to analyze the three components separately, necessitating a substantially larger sample size that would be logistically and financially unfeasible within the framework of a single-center trial. Isolating individual components would also introduce practical and ethical challenges in a population undergoing active chemotherapy, where strict behavioral compartmentalization is difficult to maintain.

Third, regarding the potential interaction between components and their acceptability and adherence, we would like to highlight that adherence to each individual component of the intervention will be systematically recorded throughout the trial. This will allow exploratory analyses of whether engagement with specific components correlates with outcomes, providing hypothesis-generating data to inform the design of future trials aimed at identifying the most active components of the multimodal intervention. These clarifications have been incorporated into the revised manuscript.

“A 10% loss to follow-up would take 214 to 192 - please take 194/0.9 and round up to allow for the loss to follow up”.

- We thank Reviewer #2 for identifying this inaccuracy. The reviewer is correct that applying a 10% loss to follow-up to the estimated sample of 194 patients requires dividing by 0.9, yielding 215.6, which should be rounded up to 216 patients rather than 214. We apologize for this error and have corrected the sample size in the revised manuscript accordingly, with 108 patients per arm for a total of 216 patients.

Additional comment for reviewer 2 regarding point #4 of the revision: “4. Have the authors described where all data underlying the findings will be made available when the study is complete?”

We would like to clarify that, as this manuscript describes a trial protocol rather than reporting study results, there are no underlying datasets to share at this time. Upon completion of the trial, de-identified participant data will be made fully available in accordance with PLOS ONE's data policy. The only restrictions on public data sharing will relate to participant privacy, as required by General Data Protection Regulation (GDPR) requirements, which is a standard and recognized exception under PLOS ONE's data policy. In the interim, all information regarding the trial protocol is publicly available at ClinicalTrials.gov (NCT07107594). This clarification has been added to the Data Availability statement in the revised manuscript.
