# Supplementary material for: Multimodal lifestyle intervention during neoadjuvant chemotherapy in patients with early breast cancer as a strategy to improve treatment response: Protocol of the DIANA randomized clinical trial
Source: PLoS One. 2026 Oct 27;21(10):e0361188. doi: 10.1371/journal.pone.0361188 (PMC13712585; doi:10.1371/journal.pone.0361188)
Supplement: Supplementary file 5 [file pone.0361188.s006.docx]

**RESPONSE TO REVIEWERS**

The authors appreciate the continued and constructive engagement of the Editor and Reviewer #2 with our protocol. We are pleased that Reviewer #1 considers the revised manuscript satisfactory. Below we provide a point-by-point response to the issues raised by Reviewer #2, together with the corresponding modifications made to the manuscript. All changes are highlighted in the “Revised Manuscript with Track Changes” file.

**General response:**

We are truly grateful to Reviewer #2 for the rigor and depth of these comments. The statistical and methodological points raised have been genuinely valuable and have, in our view, materially improved both the design and the clarity of the protocol. Before responding to each point in detail, we wish to transparently state our overall position.

We fully agree that the ideal design to answer the trial question definitively would be a large, multicenter randomized trial, powered for modest effect sizes and for ordinal and subtype-specific analyses, and equipped with long-term survival follow-up. We would welcome the chance to participate in such a study. However, the DIANA intervention is a highly structured, resource-intensive multimodal program that requires the coordinated, individualized participation of many hospital services and professionals — physiotherapists, gynecologists, oncologists, specialized nurses, psychologists, dietitians, pathologists, and radiologists — delivered throughout the entire course of neoadjuvant chemotherapy. Within our healthcare setting and with the resources currently available, a multicenter trial of this complexity is unfortunately not feasible. We have therefore sought to design the most rigorous and adequately powered single-center trial that our resources realistically permit, in order to generate solid, hypothesis-testing evidence on a question that no randomized trial has previously addressed.

The trial is, moreover, already designed, registered (ClinicalTrials.gov, NCT07107594), and in the recruitment phase, which necessarily constrains the extent to which the core design can now be modified. We have nonetheless been able to incorporate the Reviewer’s recommendations by strengthening the statistical analysis plan, by framing the effect size transparently, and by adding a concrete, pre-specified commitment to extended long-term follow-up. We hope that this combination — preserving a feasible, already-enrolling design while materially improving its analysis and its long-term interpretability — constitutes a constructive and honest response to the concerns raised.

**Response to the Reviewer’s comments:**

Reviewer #2

“I do acknowledge that RCB is an ordinal measure and not a binary one. However, the sample size makes no mention of the analysis as an ordinal outcome — there are techniques for powering ordinal regression calculations and it is unclear why this is not given here.”

- We thank Reviewer #2 for this methodological point, which has led us to strengthen and clarify the analytical framework of the trial.

The primary endpoint of DIANA was intentionally operationalized as a dichotomy — favorable pathological response (fRCB, RCB class 0–I) versus unfavorable response (uRCB, RCB class II–III) — for three reasons: first, the RCB 0–I versus II–III boundary marks the most prognostically meaningful divide in the RCB continuum, being consistently associated with divergent event-free and overall survival across breast cancer subtypes; second, a binary favorable-response rate is directly interpretable for clinical and shared decision-making; and third, this dichotomy is the endpoint on which the trial was registered (ClinicalTrials.gov, NCT07107594). Accordingly, the sample size was computed for a comparison of two proportions.

At the same time, we fully agree with the Reviewer that the RCB index is ordinal and that ordinal methods make more complete use of the four ordered response categories. We have therefore made two changes:

1. We have added a pre-specified proportional-odds (ordinal logistic regression) analysis of the full four-level RCB-class distribution (0 / I / II / III), adjusted for immunohistochemical subtype and tumor stage, as a key secondary analysis of the primary endpoint. This model yields a single common odds ratio for the shift across the ordered categories and uses the entire response distribution rather than a single cut-point. The proportional-odds assumption will be tested (score / Brant test); if it is not satisfied, a partial proportional-odds model will be reported, together with the pre-specified analysis at the fRCB/uRCB cut-point.
2. We have added an ordinal power justification to the sample size section. Using Whitehead’s method for ordinal outcomes, and an illustrative control-arm RCB-class distribution consistent with pooled data (*Yau et al., Lancet Oncol 2022*), a proportional-odds model with the same assumed common odds ratio (OR 2.25) attains greater than 85% power with the planned sample. Equivalently, the ordinal analysis retains at least 80% power to detect a smaller common odds ratio of approximately 2.07. This reflects the greater statistical efficiency of the ordinal approach.

We believe these modifications directly address the Reviewer's concern: the ordinal nature of RCB is now explicitly incorporated into both the analysis plan and the power justification, while the interpretable dichotomized endpoint is retained as the registered primary analysis. We chose to retain the dichotomous endpoint as primary, rather than building the primary sample size around the more statistically efficient ordinal model, because the trial was powered on this clinically interpretable endpoint before the Reviewer's comments, has already been approved by the competent authorities, and is already recruiting. Nonetheless, the ordinal analysis is now incorporated as a pre-specified secondary analysis that makes fuller use of the same data without altering the primary hypothesis or the recruitment plan.

“…it is not clear what a change from 40% to 60% responding favourably means in terms of the ordinal odds ratio which is what is required here; as it stands it can only be thought of as an odds ratio of 16/36 for adverse outcomes or 4/9 which seems to be a very large effect size. One would need to justify this in terms of the expected greater adherence and what this would do to response, and any mechanistic evidence that for the same dose the intervention alone will increase the potency.”

- We thank the Reviewer, and we confirm the Reviewer’s arithmetic. A shift in favorable response from 40% (control) to 60% (intervention) corresponds to an odds ratio of 2.25 for favorable response, equivalently an OR of 0.44 (that is, 4/9, or 16/36) for unfavorable response. This value is now stated explicitly in both the primary-endpoint description and the sample size section, so that the magnitude of the hypothesized effect is transparent. We wish to clarify the status of this effect size and then justify its plausibility, as the Reviewer requests.

Authors are asked to justify why this effect size represents the minimal clinically relevant difference. We interpret the 20-percentage-point difference (OR 2.25) as the target effect that the trial is powered to detect (the smallest effect that this feasibility-bounded single-center design can reliably identify with 80% power), rather than the effect we necessarily expect to observe. We consider a 20-percentage-point increase in favorable response to be clinically meaningful, as it would move approximately one in five patients into a more favorable prognostic category (RCB 0–I), which is associated with materially better long-term outcomes. In the I-SPY2 trial, estimated 3-year event-free survival by RCB class was 94% for RCB-0 (pCR) and 87% for RCB-I, compared with 80% for RCB-II and 62% for RCB-III (*JAMA Oncol 2021; 7: 1654)*, and the prognostic separation across RCB classes has since been confirmed across phenotypic subtypes in a multicenter pooled analysis of 5161 patients (*Lancet Oncol 2022;23:149–160*). These estimates indicate that the RCB 0–I versus II–III boundary that defines fRCB separates groups with substantially different long-term prognosis, and support our position that shifting approximately one in five patients across this boundary would represent a clinically relevant gain.

At the same time, we openly acknowledge that smaller improvements would also be clinically relevant; detecting such smaller effects with confidence would require a larger, multicenter trial, which, as explained in our General response, is not feasible in our setting. For this reason, we frame DIANA as a rigorous first randomized test of this intervention rather than a definitive efficacy trial, and we commit to long-term follow-up as stated in the following comments so that its data remain interpretable. We have revised the manuscript to make this framing explicit, because we agree that the previous wording was ambiguous.

The plausibility of an effect of this order is supported as follows:

- *Sensitivity of the endpoint*. fRCB (RCB 0–I) is a more sensitive endpoint than pCR alone, because it also captures clinically meaningful downshifts between adjacent RCB classes rather than only complete responses. Effective interventions have been shown to shift the entire RCB distribution toward lower values, not merely to increase pCR (*I-SPY2; JAMA Oncol 2021*). The dichotomy at RCB 0–I therefore captures a broader spectrum of benefit than a pCR endpoint would, which increases the detectable signal for a given sample size.
- *Adherence and dose–response*. The hypothesized effect is expected to be adherence-dependent. Greater engagement with the program implies a higher cumulative “dose” of exercise, nutritional optimization, and psychological support, and hence a larger anticipated modulation of the tumor microenvironment. To make this testable, adherence to each individual component will be rigorously quantified throughout the trial, and pre-specified exploratory analyses will relate cumulative intervention exposure to pathological response (a dose–response analysis).
- *Mechanistic plausibility at a fixed cytotoxic dose*. For an identical chemotherapy regimen and dose, the intervention is hypothesized to increase antitumor efficacy through several complementary and biologically supported pathways. In preclinical models of breast cancer, exercise increased tumor vascularity and perfusion, reduced intratumoral hypoxia, and improved chemotherapeutic response without any change in cytotoxic dose (*J Natl Cancer Inst 2015;107(5):djv040)*. More broadly, exercise regulates the tumor microenvironment through vascular, immune, and metabolic mechanisms, including activation of antitumor immunity and attenuation of systemic inflammation and insulin/IGF-1 signaling (*Nat Rev Cancer 2017;17:620–632*). These mechanisms provide a rationale by which the same cytotoxic dose may achieve a greater pathological response. We acknowledge that this evidence is preclinical and that its translation to the clinical setting is precisely what the DIANA trial is designed to test.
- *Comparable empirical precedent*. The most directly comparable randomized evidence, the LEANer trial (*J Clin Oncol 2023*), reported a 25-percentage-point absolute increase in pCR (53% vs 28%) with a combined exercise-and-nutrition intervention delivered during NAC; a larger effect than the 20-percentage-point difference in fRCB hypothesized here, and achieved with only pCR as the endpoint.

Finally, we have added an explicit and honest acknowledgment (Discussion, Limitations) that the true effect may be smaller than the target effect. Because the ordinal analysis is powered for a somewhat smaller common OR (2.07), and because a smaller yet clinically relevant benefit would nonetheless merit detection, we now frame confirmation of such effects as requiring a larger multicenter trial and long-term follow-up. We hope this reframing — a transparent target effect, an explicit odds ratio, a mechanistic and adherence-based rationale, and an honest statement of residual uncertainty — satisfactorily addresses the Reviewer’s concern.

“…with a complex intervention, if the trial is underpowered, or if long-term data is not collected then it is unlikely that the same trial will be run again in the case of an inconclusive result. Such a conclusion would effectively waste the clinical data here as it could not be fully interpreted. This is why a commitment to long-term follow-up is called for by patient groups and indeed the ongoing international collaborations on breast cancer clinical trial design. Strengthening this commitment would be important here…”.

- We fully agree, and we have strengthened this commitment substantially. We share the Reviewer’s concern that a short-term surrogate endpoint, without long-term data, would limit the interpretability and long-term value of the trial, and we recognize the priority that patient groups and international breast-cancer trial collaborations place on long-term follow-up. We wish to be precise about the status of this follow-up.

The trial period and its funding end at the 1-year postoperative visit, so disease-free, distant recurrence-free and overall survival are not trial endpoints and do not enter the sample size calculation. However, all participants remain under routine oncological surveillance at our institution, and we commit to recording these outcomes annually for a minimum of 5 years after surgery, with the intention to extend to 10 years, ascertained through routine clinical follow-up, institutional health records and the national mortality registry. Because it relies on care these patients receive regardless, this commitment does not depend on additional trial funding and adds no participant burden, which is what makes it sustainable. The analysis plan is stated in the manuscript (Statistical Analyses), and the follow-up will be added to the trial registration; the Ethics Committee will be consulted regarding the corresponding amendment and any required re-consent.

We thank Reviewer #2 once again. We believe the revised manuscript now explicitly incorporates the ordinal structure of RCB into both the analysis plan and the power justification; states the effect size as an odds ratio, frames it transparently as the target effect the trial is powered to detect and justifies it on adherence-related and mechanistic grounds while acknowledging residual uncertainty; and commits to a concrete, pre-specified programme of long-term follow-up. We hope these revisions address the remaining concerns and render the protocol acceptable for publication.
