# Supplementary material for: Multimodal lifestyle intervention during neoadjuvant chemotherapy in patients with early breast cancer as a strategy to improve treatment response: Protocol of the DIANA randomized clinical trial
Source: PLoS One. 2026 Oct 27;21(10):e0361188. doi: 10.1371/journal.pone.0361188 (PMC13712585; doi:10.1371/journal.pone.0361188)
Supplement: Peer review history

# Peer review: Decision Letter 0

**DOI:** 10.1371/journal.pone.0361188.r001

**Authors:** Mohamad K. Abou Chaar

22 Apr 2026

Dear Dr. Martinez-Palli,

Thank you for submitting your manuscript to PLOS ONE. After careful consideration, we feel that it has merit but does not fully meet PLOS ONE’s publication criteria as it currently stands. Therefore, we invite you to submit a revised version of the manuscript that addresses the points raised during the review process.

Please submit your revised manuscript by Jun 06 2026 11:59PM. If you will need more time than this to complete your revisions, please reply to this message or contact the journal office at plosone@plos.org. When you're ready to submit your revision, log on to https://www.editorialmanager.com/pone/ and select the 'Submissions Needing Revision' folder to locate your manuscript file.

- A letter that responds to each point raised by the academic editor and reviewer(s). You should upload this letter as a separate file labeled 'Response to Reviewers'.
- A marked-up copy of your manuscript that highlights changes made to the original version. You should upload this as a separate file labeled 'Revised Manuscript with Track Changes'.
- An unmarked version of your revised paper without tracked changes. You should upload this as a separate file labeled 'Manuscript'.

If you would like to make changes to your financial disclosure, please include your updated statement in your cover letter. Guidelines for resubmitting your figure files are available below the reviewer comments at the end of this letter.

If applicable, we recommend that you deposit your laboratory protocols in protocols.io to enhance the reproducibility of your results. Protocols.io assigns your protocol its own identifier (DOI) so that it can be cited independently in the future. For instructions see: https://journals.plos.org/plosone/s/submission-guidelines#loc-laboratory-protocols. Additionally, PLOS ONE offers an option for publishing peer-reviewed Lab Protocol articles, which describe protocols hosted on protocols.io. Read more information on sharing protocols at https://plos.org/protocols?utm_medium=editorial-email&utm_source=authorletters&utm_campaign=protocols.

As the corresponding author, your ORCID iD is verified in the submission system and will appear in the published article. PLOS supports the use of ORCID, and we encourage all coauthors to register for an ORCID iD and use it as well. Please encourage your coauthors to verify their ORCID iD within the submission system before final acceptance, as unverified ORCID iDs will not appear in the published article. *Only*  the individual author can complete the verification step; PLOS staff *cannot*  verify ORCID iDs on behalf of authors.

We look forward to receiving your revised manuscript.

Kind regards,

Mohamad K. Abou Chaar, M.D.

Academic Editor

PLOS One

Journal Requirements:

When submitting your revision, we need you to address these additional requirements.

1. Please ensure that your manuscript meets PLOS ONE's style requirements, including those for file naming. The PLOS ONE style templates can be found at

https://journals.plos.org/plosone/s/file?id=wjVg/PLOSOne_formatting_sample_main_body.pdf and

https://journals.plos.org/plosone/s/file?id=ba62/PLOSOne_formatting_sample_title_authors_affiliations.pdf.

2. Thank you for stating in your Funding Statement:

“The present work is financially supported by Fundació La Marató de TV3 and Fundació Clínic-La Pedrera. The funding sources were not involved in the study design, data collection, analysis, or interpretation, nor in the writing of the manuscript or the decision to submit it for publication. “

Please provide an amended statement that declares *all* the funding or sources of support (whether external or internal to your organization) received during this study, as detailed online in our guide for authors at http://journals.plos.org/plosone/s/submit-now.  Please also include the statement “There was no additional external funding received for this study.” in your updated Funding Statement.

Please include your amended Funding Statement within your cover letter. We will change the online submission form on your behalf.

3. We note that the grant information you provided in the ‘Funding Information’ and ‘Financial Disclosure’ sections do not match.

When you resubmit, please ensure that you provide the correct grant numbers for the awards you received for your study in the ‘Funding Information’ section.

4. Please be informed that funding information should not appear in the Acknowledgments section or other areas of your manuscript. We will only publish funding information present in the Funding Statement section of the online submission form. Please remove any funding-related text from the manuscript.

5. Your ethics statement should only appear in the Methods section of your manuscript. If your ethics statement is written in any section besides the Methods, please delete it from any other section.

6. Please include a copy of Table 1 which you refer to in your text on page 24.

7. If the reviewer comments include a recommendation to cite specific previously published works, please review and evaluate these publications to determine whether they are relevant and should be cited. There is no requirement to cite these works unless the editor has indicated otherwise.

Additional Editor Comments (if provided):

The authors present a well-structured and timely study protocol addressing an important gap in breast cancer care by rigorously evaluating a multimodal lifestyle intervention during neoadjuvant chemotherapy. I would suggest paying attention to the following points:

- Several sections use conversational wording and occasional redundancy. Consider replacing with more formal alternatives and consolidating repeated claims.

- Your statistical plan includes a log-rank test (time-to-event), but the protocol’s primary and listed outcomes are mainly cross-sectional/ordinal/categorical (RCB class, pCR, questionnaire scores, biomarkers, etc.). Please explain/clarify.

- Some inconsistency was noted. You state an “estimated 20% improvement in RCB index scores,” but later you operationalize the primary endpoint as fRCB rate improving from ~40% to 60%.

Thank you

[Note: HTML markup is below. Please do not edit.]

Reviewers' comments:

Reviewer's Responses to Questions

**Comments to the Author**

1. Does the manuscript provide a valid rationale for the proposed study, with clearly identified and justified research questions?

Reviewer #1: Yes

Reviewer #2: Partly

**********

2. Is the protocol technically sound and planned in a manner that will lead to a meaningful outcome and allow testing the stated hypotheses?

Reviewer #1: Partly

Reviewer #2: No

**********

3. Is the methodology feasible and described in sufficient detail to allow the work to be replicable?

Reviewer #1: Yes

Reviewer #2: No

**********

4. Have the authors described where all data underlying the findings will be made available when the study is complete?

The PLOS Data policy

Reviewer #1: Yes

Reviewer #2: No

**********

5. Is the manuscript presented in an intelligible fashion and written in standard English?

Reviewer #1: Yes

Reviewer #2: Yes

**********

Please use the space provided to explain your answers to the questions above and, if applicable, provide comments about issues authors must address before this protocol can be accepted for publication. You may also include additional comments for the author, including concerns about research or publication ethics.

You may also provide optional suggestions and comments to authors that they might find helpful in planning their study.

Reviewer #1: - The main outcome of the trial is supposed to be the pathological tumoral response. Although the interventions described would most likely affect tumnoral response, the problem of many different types of drugs that can be used for NAC (neoadjuvant chemotherapy), responses to which can be varied depending on the type of breast cancer being treated and the individual patient. Also there could be variations in doses, number of treatments, etc. This could be a strong confounding factor. How are the proposers planning to account for this? It is stated that randomization will be stratified according to the tumor histological subtype, which would be another confounding factor - what exactly does this "stratification" mean? The secondary outcomes may be less affected by the type of NAC except for physical variables, since different NAC would have different physical side effects.

- It is not clear when the supervised one-hour exercise sessions in the hospital gym will take place; are these sessions being done on the NAC administration day? If so, the day will be longer than usual for patients, and perhaps more difficult after administration of the NAC. However, asking patients to come to the hospital on days not scheduled for NAC might be difficult, thus limiting accrual to the trial. The same consideration exists for the psychological intervention sessions - are they held on a different day, same day as NAC? Both may prove to be difficult for patients.

- In the maintenance phase, patients will be asked to continue their exercise program at a local gym. Will gym membership be paid by the trial? What if patients don't have access to a local gym?

Reviewer #2: The authors present a multimodal complex intervention trial to improve outcomes in women receiving NAC for breast cancer. The suggestion is that this intervention will improve pathological response rates as measured by the residual burden score.

The motivation is clear but the suggested improvements are perhaps somewhat overstated especially as these are women who are more likely to die from breast cancer, and the EBCTCG results on 200,000 women published at SBCS suggest a 20% reduction is distant recurrence is going to be associated with a difference in BMI of at least 12 points. The effect on breast cancer mortality is what is important here and this may also be driven by better adherence but given that it is reliably determined that the effect on breast cancer mortality is about 2/3 of that on distant recurrence the effects discussed here are of the order of those of adjuvant chemotherapy.

The justification for stopping follow-up so soon needs to be given especially as the risk of recurrence in ER -ve disease persists for 5 years, and that for ER positive disease for 20 years. Patient groups have repeatedly stressed the importance of long-term follow-up and we do not know if response rates are a good surrogate for this particular intervention.

The effect size here to change from 60% RCB2-3 to 40% RCB2-3 needs to be justified; the associated odds ratio is 0.67 which is quite large for a short term intervention. This effect size is larger than the well known KEYNOTE trials which initially appears implausible.

The issue of receptor status is important here as this is highly prognostic and the potentially predictive nature of the marker has not been discussed in the issue of response rates - higher numbers of ER+ women with high Ki-67 may lead to lower response rates. What NAC regimens are allowed and how is the use of drugs like pembrolizumab allowed for in the randomisation/eligibility?

The intervention is complex, and it is unclear why the design does not allow the different components to be analysed separately or indeed whether any one component affects the acceptability/adherence for the others.

A 10% loss to follow-up would take 214 to 192 - please take 194/0.9 and round up to allow for the loss to follow up

**********

what does this mean?). If published, this will include your full peer review and any attached files.

If you choose “no”, your identity will remain anonymous but your review may still be made public.

**Do you want your identity to be public for this peer review?**  For information about this choice, including consent withdrawal, please see our Privacy Policy

Reviewer #1: No

Reviewer #2: No

**********

[NOTE: If reviewer comments were submitted as an attachment file, they will be attached to this email and accessible via the submission site. Please log into your account, locate the manuscript record, and check for the action link "View Attachments". If this link does not appear, there are no attachment files.]

To ensure your figures meet our technical requirements, please review our figure guidelines: https://journals.plos.org/plosone/s/figures

You may also use PLOS’s free figure tool, NAAS, to help you prepare publication quality figures: https://journals.plos.org/plosone/s/figures#loc-tools-for-figure-preparation.

NAAS will assess whether your figures meet our technical requirements by comparing each figure against our figure specifications.

# Author comment: Author response to Decision Letter 1

**DOI:** 10.1371/journal.pone.0361188.r002

25 May 2026

The authors would like to thank the Editor and the reviewers for their constructive feedback and technical checks. This letter provides a point-by-point response to all comments, along with the corresponding modifications made to the revised manuscript.

Journal requirements

In response to the journal requirements, we have carefully revised the manuscript to ensure compliance with PLOS ONE style and formatting guidelines:

- The Funding Statement has been amended to declare all sources of financial support and now includes the sentence "There was no additional external funding received for this study." Grant numbers have been verified and reconciled across the Funding Information and Financial Disclosure sections. Funding-related text has been removed from the manuscript.

- The ethics statement has been confirmed to appear exclusively within the Ethics and Dissemination subsection, which forms part of the Materials and Methods section of the manuscript.

- A copy of Table 1 (S2 Table) has been included in the revised manuscript as requested.

Response to the Editor’s comments:

“Several sections use conversational wording and occasional redundancy. Consider replacing with more formal alternatives and consolidating repeated claims”.

- We thank the Editor for this observation. We have carefully reviewed the manuscript and revised sections containing conversational wording or redundant phrasing, replacing them with more formal and concise alternatives. For instance, we replaced "Nowadays, strategies to increase response rates remain unexplored" with "Strategies to increase pathological response rates remain insufficiently explored"; replaced informal phrasing such as "it is convenient to first study" with "it is appropriate to first evaluate" in the Gender perspective section; and consolidated repeated mentions of the anticipated intervention benefits across the Discussion into a single, streamlined paragraph. All specific changes are highlighted in the revised manuscript with tracked changes.

“Your statistical plan includes a log-rank test (time-to-event), but the protocol’s primary and listed outcomes are mainly cross-sectional/ordinal/categorical (RCB class, pCR, questionnaire scores, biomarkers, etc.). Please explain/clarify”.

- We thank the Editor for identifying this inconsistency. As the Editor correctly notes, the primary and secondary outcomes of the DIANA trial are predominantly categorical, ordinal, or continuous variables, for which the log-rank test is not appropriate. The sentence has been removed from the revised manuscript and replaced with a clarification of the correct statistical approaches for each type of outcome.

“Some inconsistency was noted. You state an “estimated 20% improvement in RCB index scores,” but later you operationalize the primary endpoint as fRCB rate improving from ~40% to 60%. Thank you”

- We agree with the editor that the phrasing "estimated 20% improvement in RCB index scores" in the Abstract is imprecise and potentially ambiguous. This has been corrected in the revised manuscript to ensure consistency with the operationalization of the primary endpoint throughout the protocol. The Abstract now reads "an estimated 20-percentage point improvement in fRCB rates, from approximately 40% in the control group to 60% in the intervention group", which is consistent with the sample size calculation and the primary endpoint definition described in the Methods section.

Response to the Reviewer’s comments:

Reviewer #1

“The main outcome of the trial is supposed to be the pathological tumoral response. Although the interventions described would most likely affect tumoral response, the problem of many different types of drugs that can be used for NAC (neoadjuvant chemotherapy), responses to which can be varied depending on the type of breast cancer being treated and the individual patient. Also, there could be variations in doses, number of treatments, etc. This could be a strong confounding factor. How are the proposers planning to account for this?”

- We thank Reviewer #1 for raising this important point. We fully acknowledge that variability in NAC regimens represents a potential confounding factor, and several strategies have been incorporated into the trial design to address this. First, only patients receiving cytotoxic neoadjuvant chemotherapy will be eligible; excluding regimens based on endocrine therapy or CDK4/6 inhibitors. Second, all administered regimens will be comprehensively documented, including pharmacological agents, doses, and administration schedules. Regarding tumor subtype variability, randomization is stratified by immunohistochemical subtype (luminal-like HER2−, HER2+, and triple-negative), which ensures balanced distribution of the main subtypes — and, by extension, their associated standard regimens — across both arms. As a result, the proportion of patients receiving subtype-specific agents (e.g., pembrolizumab in triple-negative breast cancer or trastuzumab/pertuzumab in HER2-positive tumors) is expected to be comparable between groups. For the primary endpoint analysis, a multivariable logistic regression model will be performed incorporating treatment allocation, immunohistochemical subtype, and tumor stage as covariates. Since immunohistochemical subtype inherently captures both regimen-related information and intrinsic chemosensitivity differences, adjusting by subtype is more methodologically appropriate than adjusting by NAC regimen directly. The robustness of findings across different therapeutic backbones will be further assessed through pre-specified exploratory sensitivity analyses stratified by NAC treatment category (chemotherapy alone, immunotherapy-containing, and anti-HER2-containing regimens). These considerations have been clarified in the revised manuscript.

“It is stated that randomization will be stratified according to the tumor histological subtype, which would be another confounding factor. What exactly does this "stratification" mean? The secondary outcomes may be less affected by the type of NAC except for physical variables, since different NAC would have different physical side effects”.

- Stratified randomization by immunohistochemical subtype means that, at the time of randomization, patients are allocated to the intervention or control group separately within each tumoral subtype stratum (luminal-like HER2−, HER2+, and triple-negative breast cancer). This ensures that each subtype is represented in comparable proportions across both arms, regardless of the overall enrollment sequence. This stratification is particularly relevant in the context of this trial, since HER2-positive and triple-negative disease, although less prevalent in the general breast cancer population, are disproportionately represented among candidates for NAC and are associated with distinct chemotherapy regimens and response rates. By balancing tumor subtype distribution between groups, stratified randomization controls for both the biological heterogeneity of the tumor and the regimen-related variability inherent to each subtype, thereby reducing the risk of confounding in the primary endpoint analysis. Regarding the secondary outcomes, we agree with the reviewer that physical variables may be particularly susceptible to regimen-specific toxicity profiles. For this reason, all administered NAC regimen details will be systematically documented and incorporated in exploratory sensitivity analyses stratified by treatment category (chemotherapy alone, immunotherapy-containing, and anti-HER2-containing regimens), as described in the revised statistical plan.

“It is not clear when the supervised one-hour exercise sessions in the hospital gym will take place; are these sessions being done on the NAC administration day? If so, the day will be longer than usual for patients, and perhaps more difficult after administration of the NAC. However, asking patients to come to the hospital on days not scheduled for NAC might be difficult, thus limiting accrual to the trial. The same consideration exists for the psychological intervention sessions - are they held on a different day, same day as NAC? Both may prove to be difficult for patients”.

- We thank Reviewer #1 for raising this fundamental concern, which was carefully considered and discussed during the study design phase.

Rather than imposing a fixed prehabilitation schedule, the trial has been deliberately designed around a flexible, patient-centered approach to maximize adherence and comfort for patients, since the ultimate aim of the trial is to improve the treatment for breast cancer patients and their overall health.

For the exercise sessions, scheduling is discussed individually with each patient during the first physiotherapy appointment, which coincides with the baseline CPET assessment performed prior to the start of NAC, thereby combining clinical assessments and program planning in a single visit. Exercise sessions are therefore not systematically scheduled on NAC administration days, as this would be physically demanding and logistically burdensome for patients.

The same flexible approach applies to the psychological and nutritional interventions, for which an initial appointment — offered via telehealth when appropriate — is held with the corresponding specialist to agree on the most suitable schedule for each patient individually. This coordination is facilitated by continuous communication across the multidisciplinary team, including oncologists, specialized nurses, physiotherapists, psychologists, and dietitians. This clarification has been incorporated into the revised manuscript.

“In the maintenance phase, patients will be asked to continue their exercise program at a local gym. Will gym membership be paid by the trial? What if patients don't have access to a local gym?”

- We appreciate Reviewer #1 for this practical observation. We would like to clarify that gym membership costs during the maintenance phase are fully covered by the trial, so no financial burden is placed on participants. Furthermore, the local gym is located in close proximity to the hospital (approximately 450 meters, a 5-minute walk), which was deliberately chosen to minimize logistical barriers for patients and promote retention. The facility is staffed by physical trainers with specific expertise in oncological patients, ensuring that the continuity and quality of the supervised training is maintained throughout the maintenance phase. Patients who are unable to access the designated gym for medical or personal reasons will be offered to continue the maintenance phase in the hospital gym. This clarification has been incorporated into the revised manuscript.

Reviewer 2

“The authors present a multimodal complex intervention trial to improve outcomes in women receiving NAC for breast cancer. The suggestion is that this intervention will improve pathological response rates as measured by the residual burden score. The motivation is clear but the suggested improvements are perhaps somewhat overstated especially as these are women who are more likely to die from breast cancer, and the EBCTCG results on 200,000 women published at SBCS suggest a 20% reduction is distant recurrence is going to be associated with a difference in BMI of at least 12 points. The effect on breast cancer mortality is what is important here and this may also be driven by better adherence but given that it is reliably determined that the effect on breast cancer mortality is about 2/3 of that on distant recurrence the effects discussed here are of the order of those of adjuvant chemotherapy.”

- We thank Reviewer #2 for this thoughtful comment. The authors would like to clarify a fundamental distinction regarding the endpoints being discussed. The EBCTCG analysis presented at SABCS 2024 (Pan et al., Abstract GS2-09) examines the observational association between BMI at diagnosis and long-term outcomes in breast cancer patients, such as distant recurrence and breast cancer mortality. Importantly, this analysis does not evaluate the effect of BMI reduction as an intervention on these outcomes – it describes a prognostic association, not a causal interventional effect. While we fully acknowledge the considerable statistical power afforded by the large sample size and the scientific rigor of the EBCTCG group, the findings should nonetheless be interpreted with appropriate caution, given both the observational nature of the data and the fact that the work has, to date, been disseminated as a conference abstract rather than a full peer-reviewed publication. The primary endpoint of the DIANA trial is pathological tumor response at the time of surgery, as measured by the validated RCB index, a surrogate endpoint that is biologically and temporally distinct from distant recurrence or mortality. The DIANA trial is therefore not claiming a mortality benefit equivalent to that of adjuvant chemotherapy but rather hypothesizing an improvement in a short-term surrogate oncological endpoint that is potentially modifiable by biological mechanisms acting within the timeframe of NAC.

“The justification for stopping follow-up so soon needs to be given especially as the risk of recurrence in ER -ve disease persists for 5 years, and that for ER positive disease for 20 years. Patient groups have repeatedly stressed the importance of long-term follow-up and we do not know if response rates are a good surrogate for this particular intervention.”

- The authors completely agree with Reviewer #2 that long-term outcomes such as disease recurrence and mortality are of paramount clinical importance in breast cancer research, and we acknowledge that recurrence risk persists for many years, particularly in ER-positive disease. However, assessing these outcomes as primary endpoints falls outside the scope of the current trial. The primary aim of the DIANA trial is to evaluate the short-term impact of a multimodal lifestyle intervention on pathological tumor response – hypothetically mediated through modulation of the tumor microenvironment – as measured by the RCB index in the surgical specimen obtained after NAC completion. The 1-year postoperative follow-up was deliberately included to assess whether the multimodal prehabilitation program may promote sustained lifestyle changes beyond the intervention period, and to capture medium-term physical, nutritional, psychological, and quality-of-life outcomes. While recurrence and survival data will be collected at this timepoint, they are not the primary focus of the current trial. Regarding the use of RCB as a surrogate endpoint, it is worth noting that RCB is a well-validated prognostic tool with demonstrated association with long-term survival outcomes across breast cancer subtypes. We fully acknowledge, however, that whether RCB improvement translates into long-term survival benefit in the context of a lifestyle intervention specifically requires confirmation, and extended long-term follow-up of DIANA trial participants is planned as a future research priority. This has been clarified in the revised manuscript.

“The effect size here to change from 60% RCB2-3 to 40% RCB2-3 needs to be justified; the associated odds ratio is 0.67 which is quite large for a short term intervention. This effect size is larger than the well known KEYNOTE trials which initially appears implausible.”

- We thank Reviewer #2 for this methodologically important observation. We acknowledge that the proposed effect size might appear to be ambitious; however, we would like to provide a comprehensive justification supporting its plausibility from multiple angles.

First, regarding the primary endpoint itself, it is important to highlight that the RCB index represents a considerably richer and more sensitive outcome measure than pCR alone. Unlike pCR, which is a binary endpoint capturing only the presence or absence of residual disease, the RCB index quantifies the full continuum of pathological tumoral response by incorporating the two-dimensional area of residual invasive cancer, the cellularity of the tumor bed, and the nodal burden. This means that clinically meaningful improvements in tumor response – such as a shift from RCB class III to RCB class II, or from RCB class II to RCB class I – are captured and contribute to the primary endpoint, even when they do not result in a complete pathological response. The prognostic relevance of this continuum is well established: data from the I-SPY2 randomized clinical trial (JAMA Oncol. 2021 Nov 1;7(11):1654-1663, n=938) demonstrated that RCB was consistently and independently prognostic across all breast cancer subtypes and treatment categories, with event-free survival worsening significantly per unit of RCB increase in every subtype. Critically, that study also showed that effective investigational treatments shifted the entire distribution of RCB toward lower values, not only increasing pCR rates, and that this shift was associated with improved event-free survival in an exploratory analysis. The use of fRCB (RCB 0-I) as the primary endpoint in DIANA therefore captures a broader and more sensitive spectrum of treatment benefit than pCR alone, which supports the plausibility of detecting a significant effect with the proposed sample size.

Second, the baseline assumption of 40% fRCB in the control arm is deliberately conservative. Based on the pooled analysis by Yau et al. (Lancet Oncol. 2022 Jan;23(1):149-160; n=5,161), the global RCB distribution after NAC is approximately 45% RCB 0-I and 55% RCB II-III across all subtypes. Given that the DIANA population will include a significant proportion of luminal tumors, which are associated with lower chemosensitivity and pCR rates, a baseline estimate of 40% fRCB is justified and may even be an underestimate of the true proportion of unfavorable responses

Third, the most directly comparable clinical evidence supports the plausibility of the hypothesized effect size. The LEANer trial (J Clin Oncol. 2023 Dec 1;41(34):5285-5295) demonstrated a 25-percentage point increase in pCR rates (53% vs. 28%, p=0.037) in 72 breast cancer patients that underwent NAC assigned to a combined exercise and nutritional intervention, a larger effect than the 20-percentage point improvement in fRCB hypothesized in DIANA. A systematic review and meta-analysis of 30 studies on prehabilitation during neoadjuvant therapy (Support Care Cancer. 2024 Oct 28;32(11):749) further concluded that prehabilitation may improve pathological response and treatment completion compared to standard care. Additionally, the Neo-ACT trial (NCT05184582, n=712) was specifically designed to detect a 10% improvement in pCR with a prehabilitation strategy based on exercise alone during NAC, corroborating the biological plausibility of exercise-driven improvements in pathological response even with a unimodal approach. The DIANA intervention is multimodal, combining physical exercise, nutritional counseling, and psychological support, and may therefore produce synergistic effects exceeding those of any single component.

Fourth, regarding the comparison with the KEYNOTE trials, while definitely informative, this analogy is not directly applicable. Those trials evaluate the addition of pembrolizumab, a potent pharmacological immunotherapy agent acting through a specific immune checkpoint mechanism, whereas DIANA evaluates a non-pharmacological lifestyle intervention acting through multiple distinct and complementary biological pathways, including exercise-induced tumor vascular normalization and improved drug delivery, modulation of antitumor immune responses through enhanced NK and CD8+ T cell activity, and reduction of systemic inflammation and oncogenic hormonal signaling. These mechanisms are biologically plausible and supported by both preclinical and emerging clinical evidence, and their combined effect in the context of a truly multimodal intervention has not yet been evaluated in a rigorous randomized trial.

The corresponding clarifications have been made in the revised manuscript.

“The issue of receptor status is important here as this is highly prognostic and the potentially predictive nature of the marker has not been discussed in the issue of response rates - higher numbers of ER+ women with high Ki-67 may lead to lower response rates. What NAC regimens are allowed and how is the use of drugs like pembrolizumab allowed for in the randomization/eligibility?”

- We thank Reviewer #2 for raising this important point. We fully agree that receptor status is a fundamental aspect to take into consideration in our trial, since it is a key prognostic and predictive factor, and that the proportion of ER-positive tumors with high Ki-67 in the study population may influence overall response rates, given their well-known lower chemosensitivity. Regarding permitted NAC regimens, only patients receiving cytotoxic neoadjuvant chemotherapy will be eligible; regimens based exclusively on endocrine therapy or CDK4/6 inhibitors are excluded from the trial. Since the addition of targeted or immunotherapy agents is determined by tumor biology and constitutes an indispensable component of current standard of care in BC treatment, the use of pembrolizumab in triple-negative breast cancer and trastuzumab/pertuzumab in HER2-positive disease is permitted. All administered treatments will be comprehensively documented, including pharmacological agents, doses, and administration schedules. Randomization is stratified by immunohistochemical subtype (luminal-like HER2−, HER2+, and triple-negative), ensuring balanced distribution of the main subtypes — and, by extension, their associated standard regimens — across both arms. For the primary endpoint analysis, a multivariable logistic regression model will be performed incorporating treatment allocation, immunohistochemical subtype, and tumor stage as covariates. Since immunohistochemical subtype inherently captures both regimen-related information and intrinsic chemosensitivity differences, adjusting by subtype is more methodologically appropriate than adjusting by NAC regimen directly. The robustness of findings across different therapeutic backbones will be further assessed through pre-specified exploratory sensitivity analyses stratified by NAC treatment category (chemotherapy alone, immunotherapy-containing, and anti-HER2-containing regimens). These considerations have been clarified in the revised manuscript.

“The intervention is complex, and it is unclear why the design does not allow the different components to be analysed separately or indeed whether any one component affects the acceptability/adherence for the others.”

- We appreciate the methodological observation from Reviewer #2. The decision not to adopt a factorial design was deliberate and based on several well-founded considerations.

First, the primary scientific hypothesis of the DIANA trial is that the synergistic combination of physical exercise, nutritional counseling, and psychological support produces an effect on pathological tumor response that exceeds what any individual component could achieve in isolation. This is not an arbitrary choice: multimodal prehabilitation combining these three components has already demonstrated benefit across various surgical oncology fields [31, 32, 33], and is currently considered the recommended prehabilitation approach in these settings. Evidence in breast cancer, while more limited, also supports the multimodal strategy [1, 23], although no randomized trial has yet evaluated it specifically during NAC, which is precisely the gap that DIANA aims to address. Testing the multimodal intervention as a whole is therefore not a design limitation, but the deliberate and scientifically justified primary question of this trial.

Second, a factorial design would require at least four study arms to analyze the three components separately, necessitating a substantially larger sample size that would be logistically and financially unfeasible within the framework of a single-center trial. Isolating individual components would also introduce practical and ethical challenges in a population undergoing active chemotherapy, where strict behavioral compartmentalization is difficult to maintain.

Third, regarding the potential interaction between components and their acceptability and adherence, we would like to highlight that adherence to each individual component of the intervention will be systematically recorded throughout the trial. This will allow exploratory analyses of whether engagement with specific components correlates with outcomes, providing hypothesis-generating data to inform the design of future trials aimed at identifying the most active components of the multimodal intervention. These clarifications have been incorporated into the revised manuscript.

“A 10% loss to follow-up would take 214 to 192 - please take 194/0.9 and round up to allow for the loss to follow up”.

- We thank Reviewer #2 for identifying this inaccuracy. The reviewer is correct that applying a 10% loss to follow-up to the estimated sample of 194 patients requires dividing by 0.9, yielding 215.6, which should be rounded up to 216 patients rather than 214. We apologize for this error and have corrected the sample size in the revised manuscript accordingly, with 108 patients per arm for a total of 216 patients.

Additional comment for reviewer 2 regarding point #4 of the revision: “4. Have the authors described where all data underlying the findings will be made available when the study is complete?”

We would like to clarify that, as this manuscript describes a trial protocol rather than reporting study results, there are no underlying datasets to share at this time. Upon completion of the trial, de-identified participant data will be made fully available in accordance with PLOS ONE's data policy. The only restrictions on public data sharing will relate to participant privacy, as required by General Data Protection Regulation (GDPR) requirements, which is a standard and recognized exception under PLOS ONE's data policy. In the interim, all information regarding the trial protocol is publicly available at ClinicalTrials.gov (NCT07107594). This clarification has been added to the Data Availability statement in the revised manuscript.

Attachment Submitted filename: RESPONSE TO REVIEWERS.docx

# Peer review: Decision Letter 1

**DOI:** 10.1371/journal.pone.0361188.r003

**Authors:** Xingyu Xiong

8 Jul 2026

Dear Dr. Martinez-Palli,

Thank you for submitting your manuscript to PLOS ONE. After careful consideration, we feel that it has merit but does not fully meet PLOS ONE’s publication criteria as it currently stands. Therefore, we invite you to submit a revised version of the manuscript that addresses the points raised during the review process.

Please submit your revised manuscript by Aug 22 2026 11:59PM. If you will need more time than this to complete your revisions, please reply to this message or contact the journal office at plosone@plos.org. When you're ready to submit your revision, log on to https://www.editorialmanager.com/pone/ and select the 'Submissions Needing Revision' folder to locate your manuscript file.

- A letter that responds to each point raised by the academic editor and reviewer(s). You should upload this letter as a separate file labeled 'Response to Reviewers'.
- A marked-up copy of your manuscript that highlights changes made to the original version. You should upload this as a separate file labeled 'Revised Manuscript with Track Changes'.
- An unmarked version of your revised paper without tracked changes. You should upload this as a separate file labeled 'Manuscript'.

If you would like to make changes to your financial disclosure, please include your updated statement in your cover letter. Guidelines for resubmitting your figure files are available below the reviewer comments at the end of this letter.

If applicable, we recommend that you deposit your laboratory protocols in protocols.io to enhance the reproducibility of your results. Protocols.io assigns your protocol its own identifier (DOI) so that it can be cited independently in the future. For instructions see: https://journals.plos.org/plosone/s/submission-guidelines#loc-laboratory-protocols. Additionally, PLOS ONE offers an option for publishing peer-reviewed Lab Protocol articles, which describe protocols hosted on protocols.io. Read more information on sharing protocols at https://plos.org/protocols?utm_medium=editorial-email&utm_source=authorletters&utm_campaign=protocols.

As the corresponding author, your ORCID iD is verified in the submission system and will appear in the published article. PLOS supports the use of ORCID, and we encourage all coauthors to register for an ORCID iD and use it as well. Please encourage your coauthors to verify their ORCID iD within the submission system before final acceptance, as unverified ORCID iDs will not appear in the published article. *Only*  the individual author can complete the verification step; PLOS staff *cannot*  verify ORCID iDs on behalf of authors.

We look forward to receiving your revised manuscript.

Kind regards,

Xingyu Xiong, Ph.D.

Academic Editor

PLOS One

Journal Requirements:

If the reviewer comments include a recommendation to cite specific previously published works, please review and evaluate these publications to determine whether they are relevant and should be cited. There is no requirement to cite these works unless the editor has indicated otherwise.

Additional Editor Comments (if provided):

**Editor Decision** ：Major Revision

[Note: HTML markup is below. Please do not edit.]

Reviewers' comments:

Reviewer's Responses to Questions

**Comments to the Author**

1. Does the manuscript provide a valid rationale for the proposed study, with clearly identified and justified research questions?

Reviewer #1: Yes

Reviewer #2: Yes

**********

2. Is the protocol technically sound and planned in a manner that will lead to a meaningful outcome and allow testing the stated hypotheses?

Reviewer #1: Yes

Reviewer #2: Partly

**********

3. Is the methodology feasible and described in sufficient detail to allow the work to be replicable?

Reviewer #1: Yes

Reviewer #2: No

**********

4. Have the authors described where all data underlying the findings will be made available when the study is complete?

The PLOS Data policy

Reviewer #1: Yes

Reviewer #2: Yes

**********

5. Is the manuscript presented in an intelligible fashion and written in standard English?

Reviewer #1: Yes

Reviewer #2: Yes

**********

Please use the space provided to explain your answers to the questions above and, if applicable, provide comments about issues authors must address before this protocol can be accepted for publication. You may also include additional comments for the author, including concerns about research or publication ethics.

You may also provide optional suggestions and comments to authors that they might find helpful in planning their study.

Reviewer #1: The authors have answered and revvised satisfactorily all of the comments sent in previous review. The added text and revised text clarify previous comments.

Reviewer #2: Thank you for you response to my previous comments.

I do acknowledge that RCB is an ordinal measure and not a binary one. However, the sample size makes no mention of the analysis as an ordinal outcome - there are techniques for powering ordinal regression calculations and it is unclear why this is not given here; and it is not clear what a change from 40% to 60% responding favourably means in terms of the ordinal odds ratio which is what is required here; as it stands it can only be thought of as an odds ratio of 16/36 for adverse outcomes or 4/9 which seems to be a very large effect size. One would need to justify this in terms of the expected greater adherence and what this would do to response, and any mechanistic evidence that for the same dose the intervention alone will increase the potency.

The issue is that with a complex intervention, if the trial is underpowered, or if long-term data is not collected then it is unlikely that the same trial will be run again in the case of an inconclusive result. Such a conclusion would effectively waste the clinical data here as it could not be fully interpreted. This is why a commitment to long-term follow-up is called for by patient groups and indeed the ongoing international collaborations on breast cancer clinical trial design. Strengthening this commitment would be important here as would a very clear calculation of why the effect size is the minimal clinically relevant difference and what it actually means in terms of the analysis proposed.

**********

what does this mean?). If published, this will include your full peer review and any attached files.

If you choose “no”, your identity will remain anonymous but your review may still be made public.

**Do you want your identity to be public for this peer review?**  For information about this choice, including consent withdrawal, please see our Privacy Policy

Reviewer #1: No

Reviewer #2: No

**********

[NOTE: If reviewer comments were submitted as an attachment file, they will be attached to this email and accessible via the submission site. Please log into your account, locate the manuscript record, and check for the action link "View Attachments". If this link does not appear, there are no attachment files.]

To ensure your figures meet our technical requirements, please review our figure guidelines: https://journals.plos.org/plosone/s/figures

You may also use PLOS’s free figure tool, NAAS, to help you prepare publication quality figures: https://journals.plos.org/plosone/s/figures#loc-tools-for-figure-preparation.

NAAS will assess whether your figures meet our technical requirements by comparing each figure against our figure specifications.

# Author comment: Author response to Decision Letter 2

**DOI:** 10.1371/journal.pone.0361188.r004

17 Jul 2026

RESPONSE TO REVIEWERS

The authors appreciate the continued and constructive engagement of the Editor and Reviewer #2 with our protocol. We are pleased that Reviewer #1 considers the revised manuscript satisfactory. Below we provide a point-by-point response to the issues raised by Reviewer #2, together with the corresponding modifications made to the manuscript. All changes are highlighted in the “Revised Manuscript with Track Changes” file.

General response:

We are truly grateful to Reviewer #2 for the rigor and depth of these comments. The statistical and methodological points raised have been genuinely valuable and have, in our view, materially improved both the design and the clarity of the protocol. Before responding to each point in detail, we wish to transparently state our overall position.

We fully agree that the ideal design to answer the trial question definitively would be a large, multicenter randomized trial, powered for modest effect sizes and for ordinal and subtype-specific analyses, and equipped with long-term survival follow-up. We would welcome the chance to participate in such a study. However, the DIANA intervention is a highly structured, resource-intensive multimodal program that requires the coordinated, individualized participation of many hospital services and professionals — physiotherapists, gynecologists, oncologists, specialized nurses, psychologists, dietitians, pathologists, and radiologists — delivered throughout the entire course of neoadjuvant chemotherapy. Within our healthcare setting and with the resources currently available, a multicenter trial of this complexity is unfortunately not feasible. We have therefore sought to design the most rigorous and adequately powered single-center trial that our resources realistically permit, in order to generate solid, hypothesis-testing evidence on a question that no randomized trial has previously addressed.

The trial is, moreover, already designed, registered (ClinicalTrials.gov, NCT07107594), and in the recruitment phase, which necessarily constrains the extent to which the core design can now be modified. We have nonetheless been able to incorporate the Reviewer’s recommendations by strengthening the statistical analysis plan, by framing the effect size transparently, and by adding a concrete, pre-specified commitment to extended long-term follow-up. We hope that this combination — preserving a feasible, already-enrolling design while materially improving its analysis and its long-term interpretability — constitutes a constructive and honest response to the concerns raised.

Response to the Reviewer’s comments:

Reviewer #2

“I do acknowledge that RCB is an ordinal measure and not a binary one. However, the sample size makes no mention of the analysis as an ordinal outcome — there are techniques for powering ordinal regression calculations and it is unclear why this is not given here.”

- We thank Reviewer #2 for this methodological point, which has led us to strengthen and clarify the analytical framework of the trial.

The primary endpoint of DIANA was intentionally operationalized as a dichotomy — favorable pathological response (fRCB, RCB class 0–I) versus unfavorable response (uRCB, RCB class II–III) — for three reasons: first, the RCB 0–I versus II–III boundary marks the most prognostically meaningful divide in the RCB continuum, being consistently associated with divergent event-free and overall survival across breast cancer subtypes; second, a binary favorable-response rate is directly interpretable for clinical and shared decision-making; and third, this dichotomy is the endpoint on which the trial was registered (ClinicalTrials.gov, NCT07107594). Accordingly, the sample size was computed for a comparison of two proportions.

At the same time, we fully agree with the Reviewer that the RCB index is ordinal and that ordinal methods make more complete use of the four ordered response categories. We have therefore made two changes:

1. We have added a pre-specified proportional-odds (ordinal logistic regression) analysis of the full four-level RCB-class distribution (0 / I / II / III), adjusted for immunohistochemical subtype and tumor stage, as a key secondary analysis of the primary endpoint. This model yields a single common odds ratio for the shift across the ordered categories and uses the entire response distribution rather than a single cut-point. The proportional-odds assumption will be tested (score / Brant test); if it is not satisfied, a partial proportional-odds model will be reported, together with the pre-specified analysis at the fRCB/uRCB cut-point.

2. We have added an ordinal power justification to the sample size section. Using Whitehead’s method for ordinal outcomes, and an illustrative control-arm RCB-class distribution consistent with pooled data (Yau et al., Lancet Oncol 2022), a proportional-odds model with the same assumed common odds ratio (OR 2.25) attains greater than 85% power with the planned sample. Equivalently, the ordinal analysis retains at least 80% power to detect a smaller common odds ratio of approximately 2.07. This reflects the greater statistical efficiency of the ordinal approach.

We believe these modifications directly address the Reviewer's concern: the ordinal nature of RCB is now explicitly incorporated into both the analysis plan and the power justification, while the interpretable dichotomized endpoint is retained as the registered primary analysis. We chose to retain the dichotomous endpoint as primary, rather than building the primary sample size around the more statistically efficient ordinal model, because the trial was powered on this clinically interpretable endpoint before the Reviewer's comments, has already been approved by the competent authorities, and is already recruiting. Nonetheless, the ordinal analysis is now incorporated as a pre-specified secondary analysis that makes fuller use of the same data without altering the primary hypothesis or the recruitment plan.

“…it is not clear what a change from 40% to 60% responding favourably means in terms of the ordinal odds ratio which is what is required here; as it stands it can only be thought of as an odds ratio of 16/36 for adverse outcomes or 4/9 which seems to be a very large effect size. One would need to justify this in terms of the expected greater adherence and what this would do to response, and any mechanistic evidence that for the same dose the intervention alone will increase the potency.”

- We thank the Reviewer, and we confirm the Reviewer’s arithmetic. A shift in favorable response from 40% (control) to 60% (intervention) corresponds to an odds ratio of 2.25 for favorable response, equivalently an OR of 0.44 (that is, 4/9, or 16/36) for unfavorable response. This value is now stated explicitly in both the primary-endpoint description and the sample size section, so that the magnitude of the hypothesized effect is transparent. We wish to clarify the status of this effect size and then justify its plausibility, as the Reviewer requests.

Authors are asked to justify why this effect size represents the minimal clinically relevant difference. We interpret the 20-percentage-point difference (OR 2.25) as the target effect that the trial is powered to detect (the smallest effect that this feasibility-bounded single-center design can reliably identify with 80% power), rather than the effect we necessarily expect to observe. We consider a 20-percentage-point increase in favorable response to be clinically meaningful, as it would move approximately one in five patients into a more favorable prognostic category (RCB 0–I), which is associated with materially better long-term outcomes. In the I-SPY2 trial, estimated 3-year event-free survival by RCB class was 94% for RCB-0 (pCR) and 87% for RCB-I, compared with 80% for RCB-II and 62% for RCB-III (JAMA Oncol 2021; 7: 1654), and the prognostic separation across RCB classes has since been confirmed across phenotypic subtypes in a multicenter pooled analysis of 5161 patients (Lancet Oncol 2022;23:149–160). These estimates indicate that the RCB 0–I versus II–III boundary that defines fRCB separates groups with substantially different long-term prognosis, and support our position that shifting approximately one in five patients across this boundary would represent a clinically relevant gain.

At the same time, we openly acknowledge that smaller improvements would also be clinically relevant; detecting such smaller effects with confidence would require a larger, multicenter trial, which, as explained in our General response, is not feasible in our setting. For this reason, we frame DIANA as a rigorous first randomized test of this intervention rather than a definitive efficacy trial, and we commit to long-term follow-up as stated in the following comments so that its data remain interpretable. We have revised the manuscript to make this framing explicit, because we agree that the previous wording was ambiguous.

The plausibility of an effect of this order is supported as follows:

· Sensitivity of the endpoint. fRCB (RCB 0–I) is a more sensitive endpoint than pCR alone, because it also captures clinically meaningful downshifts between adjacent RCB classes rather than only complete responses. Effective interventions have been shown to shift the entire RCB distribution toward lower values, not merely to increase pCR (I-SPY2; JAMA Oncol 2021). The dichotomy at RCB 0–I therefore captures a broader spectrum of benefit than a pCR endpoint would, which increases the detectable signal for a given sample size.

· Adherence and dose–response. The hypothesized effect is expected to be adherence-dependent. Greater engagement with the program implies a higher cumulative “dose” of exercise, nutritional optimization, and psychological support, and hence a larger anticipated modulation of the tumor microenvironment. To make this testable, adherence to each individual component will be rigorously quantified throughout the trial, and pre-specified exploratory analyses will relate cumulative intervention exposure to pathological response (a dose–response analysis).

· Mechanistic plausibility at a fixed cytotoxic dose. For an identical chemotherapy regimen and dose, the intervention is hypothesized to increase antitumor efficacy through several complementary and biologically supported pathways. In preclinical models of breast cancer, exercise increased tumor vascularity and perfusion, reduced intratumoral hypoxia, and improved chemotherapeutic response without any change in cytotoxic dose (J Natl Cancer Inst 2015;107(5):djv040). More broadly, exercise regulates the tumor microenvironment through vascular, immune, and metabolic mechanisms, including activation of antitumor immunity and attenuation of systemic inflammation and insulin/IGF-1 signaling (Nat Rev Cancer 2017;17:620–632). These mechanisms provide a rationale by which the same cytotoxic dose may achieve a greater pathological response. We acknowledge that this evidence is preclinical and that its translation to the clinical setting is precisely what the DIANA trial is designed to test.

· Comparable empirical precedent. The most directly comparable randomized evidence, the LEANer trial (J Clin Oncol 2023), reported a 25-percentage-point absolute increase in pCR (53% vs 28%) with a combined exercise-and-nutrition intervention delivered during NAC; a larger effect than the 20-percentage-point difference in fRCB hypothesized here, and achieved with only pCR as the endpoint.

Finally, we have added an explicit and honest acknowledgment (Discussion, Limitations) that the true effect may be smaller than the target effect. Because the ordinal analysis is powered for a somewhat smaller common OR (2.07), and because a smaller yet clinically relevant benefit would nonetheless merit detection, we now frame confirmation of such effects as requiring a larger multicenter trial and long-term follow-up. We hope this reframing — a transparent target effect, an explicit odds ratio, a mechanistic and adherence-based rationale, and an honest statement of residual uncertainty — satisfactorily addresses the Reviewer’s concern.

“…with a complex intervention, if the trial is underpowered, or if long-term data is not collected then it is unlikely that the same trial will be run again in the case of an inconclusive result. Such a conclusion would effectively waste the clinical data here as it could not be fully interpreted. This is why a commitment to long-term follow-up is called for by patient groups and indeed the ongoing international collaborations on breast cancer clinical trial design. Strengthening this commitment would be important here…”.

- We fully agree, and we have strengthened this commitment substantially. We share the Reviewer’s concern that a short-term surrogate endpoint, without long-term data, would limit the interpretability and long-term value of the trial, and we recognize the priority that patient groups and international breast-cancer trial collaborations place on long-term follow-up. We wish to be precise about the status of this follow-up.

The trial period and its funding end at the 1-year postoperative visit, so disease-free, distant recurrence-free and overall survival are not trial endpoints and do not enter the sample size calculation. However, all participants remain under routine oncological surveillance at our institution, and we commit to recording these outcomes annually for a minimum of 5 years after surgery, with the intention to extend to 10 years, ascertained through routine clinical follow-up, institutional health records and the national mortality registry. Because it relies on care these patients receive regardless, this commitment does not depend on additional trial funding and adds no participant burden, which is what makes it sustainable. The analysis plan is stated in the manuscript (Statistical Analyses), and the follow-up will be added to the trial registration; the Ethics Committee will be consulted regarding the corresponding amendment and any required re-consent.

We thank Reviewer #2 once again. We believe the revised manuscript now explicitly incorporates the ordinal structure of RCB into both the analysis plan and the power justification; states the effect size as an odds ratio, frames it transparently as the target effect the trial is powered to detect and justifies it on adherence-related and mechanistic grounds while acknowledging residual uncertainty; and commits to a concrete, pre-specified programme of long-term follow-up. We hope these revisions address the remaining concerns and render the protocol acceptable for publication.

Attachment Submitted filename: RESPONSE_TO_REVIEWERS_auresp_2.docx

# Peer review: Decision Letter 2

**DOI:** 10.1371/journal.pone.0361188.r005

**Authors:** Khalid Said Mohammad

7 Oct 2026

Multimodal lifestyle intervention during neoadjuvant chemotherapy in patients with early-breast cancer as a strategy to improve treatment response: protocol of the DIANA Randomized Clinical Trial.

PONE-D-26-09274R2

Dear Dr. Martinez-Palli,

We’re pleased to inform you that your manuscript has been judged scientifically suitable for publication and will be formally accepted for publication once it meets all outstanding technical requirements.

Within one week, you’ll receive an e-mail detailing the required amendments. When these have been addressed, you’ll receive a formal acceptance letter and your manuscript will be scheduled for publication.

An invoice will be generated when your article is formally accepted. Please note, if your institution has a publishing partnership with PLOS and your article meets the relevant criteria, all or part of your publication costs will be covered. Please make sure your user information is up-to-date by logging into Editorial Manager at Editorial Manager® and clicking the ‘Update My Information' link at the top of the page. For questions related to billing, please contact billing support.

If your institution or institutions have a press office, please notify them about your upcoming paper to help maximize its impact. If they’ll be preparing press materials, please inform our press team as soon as possible -- no later than 48 hours after receiving the formal acceptance. Your manuscript will remain under strict press embargo until 2 pm Eastern Time on the date of publication. For more information, please contact onepress@plos.org.

Kind regards,

Khalid Said Mohammad

Academic Editor

PLOS One

Additional Editor Comments (optional):

Reviewers' comments:

Reviewer's Responses to Questions

**Comments to the Author**

1. Does the manuscript provide a valid rationale for the proposed study, with clearly identified and justified research questions?

Reviewer #1: Yes

Reviewer #2: Yes

**********

2. Is the protocol technically sound and planned in a manner that will lead to a meaningful outcome and allow testing the stated hypotheses?

Reviewer #1: Yes

Reviewer #2: Yes

**********

3. Is the methodology feasible and described in sufficient detail to allow the work to be replicable?

Reviewer #1: Yes

Reviewer #2: Yes

**********

4. Have the authors described where all data underlying the findings will be made available when the study is complete?

The PLOS Data policy

Reviewer #1: Yes

Reviewer #2: Yes

**********

5. Is the manuscript presented in an intelligible fashion and written in standard English?

Reviewer #1: Yes

Reviewer #2: Yes

**********

Please use the space provided to explain your answers to the questions above and, if applicable, provide comments about issues authors must address before this protocol can be accepted for publication. You may also include additional comments for the author, including concerns about research or publication ethics.

You may also provide optional suggestions and comments to authors that they might find helpful in planning their study.

Reviewer #1: All of my concerns have been previously met and explained. This re-review concerned statistical questions raised by Reviewer #2. I am satisfied with the changes made previously and with the new changes made in response to Reviewer #2.

Reviewer #2: thank you for your response to my comments.

**********

what does this mean?). If published, this will include your full peer review and any attached files.

If you choose “no”, your identity will remain anonymous but your review may still be made public.

**Do you want your identity to be public for this peer review?**  For information about this choice, including consent withdrawal, please see our Privacy Policy

Reviewer #1: No

Reviewer #2: No

**********

# Editor report: Acceptance letter

**DOI:** 10.1371/journal.pone.0361188.r006

**Authors:** Khalid Said Mohammad

PONE-D-26-09274R2

PLOS One

Dear Dr. Martinez-Palli,

I'm pleased to inform you that your manuscript has been deemed suitable for publication in PLOS One. Congratulations! Your manuscript is now being handed over to our production team.

At this stage, our production department will prepare your paper for publication. This includes ensuring the following:

* All references, tables, and figures are properly cited

* All relevant supporting information is included in the manuscript submission,

* There are no issues that prevent the paper from being properly typeset

You will receive further instructions from the production team, including instructions on how to review your proof when it is ready. Please keep in mind that we are working through a large volume of accepted articles, so please give us a few days to review your paper and let you know the next and final steps.

Lastly, if your institution or institutions have a press office, please let them know about your upcoming paper now to help maximize its impact. If they'll be preparing press materials, please inform our press team within the next 48 hours. Your manuscript will remain under strict press embargo until 2 pm Eastern Time on the date of publication. For more information, please contact onepress@plos.org.

You will receive an invoice from PLOS for your publication fee after your manuscript has reached the completed accept phase. If you receive an email requesting payment before acceptance or for any other service, this may be a phishing scheme. Learn how to identify phishing emails and protect your accounts at https://explore.plos.org/phishing.

If we can help with anything else, please email us at customercare@plos.org.

Thank you for submitting your work to PLOS One and supporting open access.

Kind regards,

PLOS One Editorial Office Staff

on behalf of

Dr. Khalid Said Mohammad

Academic Editor

PLOS One
